# *Molineria recurvata* Ameliorates Streptozotocin-Induced Diabetic Nephropathy through Antioxidant and Anti-Inflammatory Pathways

**DOI:** 10.3390/molecules27154985

**Published:** 2022-08-05

**Authors:** Prasanta Dey, Amit Kundu, Ha Eun Lee, Babli Kar, Vineet Vishal, Suvakanta Dash, In Su Kim, Tejendra Bhakta, Hyung Sik Kim

**Affiliations:** 1 School of Pharmacy, Sungkyunkwan University, 2066, Seobu-ro, Jangan-gu, Suwon 16419, Korea; 2 Bengal Homoeopathic Medical College and Hospital, Asansol 713301, India; 3 Department of Botany, Bangabasi Evening College, Kolkata 700009, India; 4 Regional Institute of Pharmaceutical Science & Technology, Agartala 799006, India

**Keywords:** *Molineria recurvata*, diabetic nephropathy, urinary biomarkers, inflammation, oxidative stress

## Abstract

*Molineria recurvata* (MR) has been traditionally used to manage diabetes mellitus in India. However, the molecular mechanism of MR on the diabetic-induced nephropathy has not been clearly investigated. Thus, this study investigates the protective effects of the MR extract on nephropathy in streptozotocin (STZ)-induced diabetic rats. Diabetes was instigated by a single intraperitoneal injection of STZ (45 mg/kg) in male Sprague-Dawley rats. Once the diabetes was successfully induced, the MR extract (200 mg/kg/day) or metformin (200 mg/kg/day) was orally administered for 14 days. Renal function, morphology changes and levels of inflammatory cytokines were measured. Blood glucose concentrations were considerably reduced in STZ-induced diabetic rats following treatment with the MR extract. The administration of the MR extract substantially restored the abnormal quantity of the oxidative DNA damage marker 8-hydroxy-2′-deoxy-guanosine (8-OHdG), malondialdehyde, glutathione, oxidized glutathione, superoxide dismutase, catalase, interleukin (IL)-1β, IL-6, IL-10, and transforming growth factor-β (TGF-β). The urinary excretion of kidney injury molecule-1 (KIM-1), neutrophil gelatinase-associated lipocalin (NGAL), selenium binding protein 1 (SBP1), and pyruvate kinase M2 (PKM2) was significantly reduced in diabetes rats after administration of the MR extracts. In the kidneys of STZ-induced diabetic rats, the MR extracts markedly downregulated the expression of fibronectin, collagen-1, and α-smooth muscle actin (α-SMA). In particular, the MR extracts markedly increased the level of SIRT1 and SIRT3 and reduced claudin-1 in the kidney. These results suggest that the MR extracts exhibits therapeutic activity in contrast to renal injury in STZ-induced diabetic rats through repressing inflammation and oxidative stress.

## 1. Introduction

Diabetic patients have developed different microvascular disorders including nephropathy, retinopathy, and neuropathy [1,2]. Around 40% of newly diagnosed patients with diabetes in both Asian and Western countries have diabetic nephropathy (DN), which is one of the major causes of end-stage renal disease (ESRD) [3]. Hypertension, obesity, and sustained hyperglycemia are the major risk factors for the initiation or progression of DN and induce glomerular malfunction and kidney damage. Since the progression of DN to ESRD is irreversible, it is important to take precise therapeutic approaches to ameliorate kidney injury. Progress has been made in our understanding of the pharmacology of DN, and effective drugs are currently being introduced for the treatment of DN in diabetic patients [4,5].

In patients with diabetes, the accumulation of advanced glycation end products (AGEs), which play a major role in the development of diabetic nephropathy, is one of the main causes of chronic kidney damage [6]. Oxidative stress and chronic inflammation are strongly connected with the development and progression of diabetic nephropathy [7]. The high excretion of urinary microalbumin, thickening of the basement membrane, and mesangial expansion are some of the primary characteristics of diabetic nephropathy [8]. The deposition of extracellular matrix (ECM) proteins also has an important role in the progression of diabetic nephropathy [9,10]. Diabetic nephropathy can also lead to glomerulosclerosis and renal fibrosis, reflecting the fact that that the epithelial-mesenchymal transition (EMT) occurs owing to the loss of E-cadherin and the deposition of α-smooth muscle actin (α-SMA) [11,12]. It has been also established that expression of transforming growth factor-β (TGF-β) is highly upregulated and glomerular function capacity is reduced in patients with diabetic nephropathy [10,13].

The plant *Molineria recurvata* (of the family Hypoxidaceae) is grown widely in Tripura and other hotter regions of India. The leaves, which have fewer or no side effects compared to marketed drugs for diabetes, have been utilized for the management of diabetic mellitus for many years in the Northeastern part of India. The leaf extract of *Molineria recurvata* (MR) possesses anticoagulant and anthelmintic activity [14,15], but the exact molecular mechanism underlying the protective effects against DN is still undetermined. Here, we aimed to evaluate the protective impacts of the MR extract on DN in streptozotocin (STZ)-induced diabetic rats. This study will also highlight the underlying molecular mechanism modulated by the MR extract on STZ-induced diabetic rats.

## 2. Results

### 2.1. Phytochemical Properties of the MR Extract

A yield of 12.01% MR methanolic extract was obtained from 100 g of leaves. As indicated in Table 1, initially phytochemical examination discovered the existence of alkaloids, carbohydrates, steroids, flavonoids, and hydroxy-anthraquinone glycosides in the methanolic leaf extract of MR [16].

### 2.2. Acute Toxicity Study of the MR Extract

We measured the toxicity of the MR extract in rats. No indications of any unusual emaciation, respiratory depression, behavior, posture, or mortality at a highest dosage (1000 mg/kg) of the MR extract were observed. No mortality was observed in the groups treated with the MR extract. Therefore, we decided 1000 mg/kg was safe to conduct our further study. Here, based on the solubility, volume, and safety of the MR extract that could be simply dispensed orally, we selected 200 mg/kg as an efficient dose.

### 2.3. Protective Impact of the MR Extract on Blood Glucose Concentrations in STZ-Administered Rats

**Figure 1:**
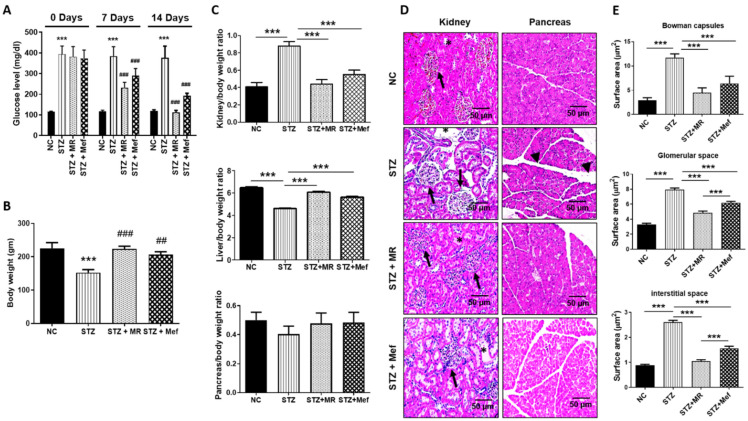
Effects of the MR extract on blood glucose level, body and organ weight, and histopathological modifications in STZ-treated rats. (**A**) Blood glucose level (fasting). (**B**) Changes in body weight. (**C**) Relative change of the kidney, liver, and pancreas weight. The abnormal alterations in these factors were restored by MR treatment. All values are the mean ± S.D. (*n* = 6). Statistical calculation was completed by one-way ANOVA subsequently by Tukey’s honest significant difference (HSD) post hoc test for multiple comparisons. (*** *p* < 0.001). ## *p* < 0.01, ### *p* < 0.001 compared with the STZ group. (**D**) Alteration of the histopathology in the kidney and pancreas (H&E stained). The STZ-treated diabetic rats displayed enlarged cortex with expansion (asterisk), glomerular sclerosis (arrowheads), and dilatation. The medulla showed interstitial nodular sclerosis, tubular dilatation, and fibroplasia (asterisk). The treatment of STZ-treated rats with the MR extract induced minor occurrence of the medulla and tubular injury, normal-sized renal cortex, and normal histological structure of thin tubules (arrows). Original magnification: 200×, scale bar: 50 μm. In contrast, the pancreas is composed of exocrine components that are tightly packed by acinar cells and organized into small lobules divided by intact intralobular and interlobular connective tissue septa. The pathology of both exocrine and endocrine components has changed after STZ treatment. Most of the acinar cells were enlarged, and tiny vacuoles were noted. The interlobular ducts were covered with a flattened epithelium (black arrowheads). STZ treatment destroyed almost all the islet β-cells. However, the MR extract restored the general pathology of the pancreas of diabetic rats. Atrophic changes in the acinar cells were negligible. The border between the exocrine and endocrine portions became more distinctive. All images are symbolic of three rats per investigational group. Original magnification: 200×, scale bar: 100 μm. (**E**) Quantitative investigation of the Bowman capsule size and glomerular and interstitial spaces of H&E-stained kidney sections in the experimental rats. NC: Normal control, STZ: streptozotocin-treated group, STZ + MR: Streptozotocin-treated rats received MR extract, STZ + Mef: Streptozotocin-treated rats received Metformin.

The oral glucose tolerance test and insulin tolerance test was initially performed in different groups of rats (Supplementary Figure S1A,B). In STZ-administered rats, the blood glucose concentration was significantly enhanced compared with the control group. After 14 days of oral administration of the MR extract and standard compounds, the fasting blood glucose amount was noticeably reduced in STZ-treated rats treated by the MR extract (200 mg/kg), to a level that was comparable with that achieved in Mef (200 mg/kg)-treated rats. Thus, it was shown that the MR extract could successfully synchronize the blood glucose amounts in STZ-stimulated diabetic rats (Figure 1A).

### 2.4. Protective Effect of the MR Extract on Organ and Body Weight Alterations in STZ-Administered Rats

The total body weight of STZ-administered rats decreased drastically contrasted to that of the normal control group. After 14 days of oral administration with the MR extract, the body weight of diabetic rats treated with the MR extract (200 mg/kg) was normalized to levels comparable to those of rats treated with Mef (200 mg/kg; Figure 1B). Additionally, the average weight of the major organs (the liver and pancreas) was boosted distinctly in STZ-administered rats after administration of the MR extract, but the MR extract caused a marked reduction in kidney weight (Figure 1C).

### 2.5. Protective Effect of the MR Extract on Histopathological Injury in STZ-Administered Rats

The histopathology of the kidney tissues was examined by Hematoxylin & eosin staining. In STZ-administered rats, both the kidneys showed distinct hydropic variation in the proximal tubules and enlarged glomerular size. Moreover, morphological changes in the interstitial space and Bowman capsules were observed, and glomerular space was enhanced in STZ-administered diabetic rats (Figure 1D,E). Nonetheless, the deformity in the kidney morphology was restored markedly after the MR extract or Mef administration (Figure 1D,E).

Moreover, a histological inspection was also performed to estimate any changes in the pancreatic acini and islets of Langerhans. STZ-administered rats exhibited few islets with numerous vacuous areas; further, they had randomly shaped islets in the pancreas which are comparable with the transparent and round borders across the islets in the pancreas of normal rats. These malformations were reestablished successfully after administration with the MR extract (200 mg/kg) or Mef (200 mg/kg), proving that the MR extract successfully recovered STZ-induced pancreatic damage (Figure 1D). Therefore, the MR extract could repair diabetes-induced damage to the pancreatic structure.

### 2.6. Protective Effect of the MR Extract on Renal Injury Biomarkers in STZ-Administered Rats

**Figure 2:**
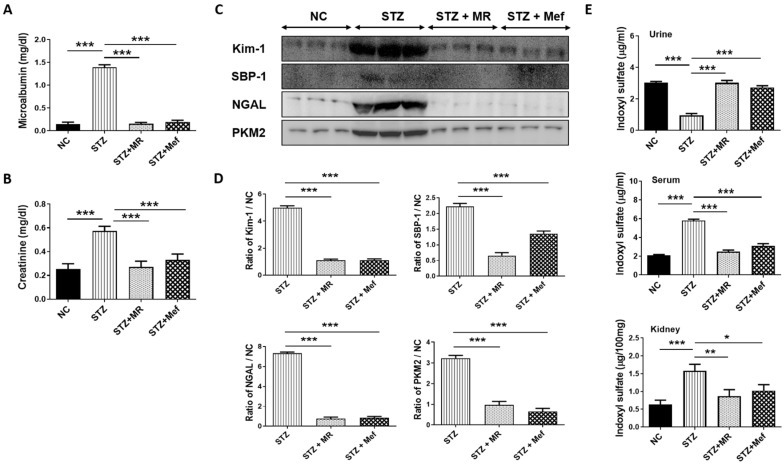
Effects of MR extract administration on biochemical and urinary parameters in STZ-induced diabetic rats. Alteration of (**A**) Urinary excretion of microalbumin and (**B**) serum creatinine level. Values are the mean ± S.D. (*n* = 6). Statistical evaluation was achieved by one-way ANOVA subsequently by Tukey’s HSD post hoc test for multiple comparisons (*** *p* < 0.001). (**C**) The expression pattern of kidney damage biomarkers (KIM-1, SBP-1, NGAL, and PKM2) in the urine. (**D**) The band intensity was evaluated densitometrically using ImageJ software (*** *p* < 0.001). (**E**) Alteration of 3-indoxyl sulfate concentration in STZ-treated rats in the urine, serum, and kidney tissues was quantified by high-performance liquid chromatography (HPLC). Values are the mean ± S.D. (*n* = 6). Statistical evaluation was completed by one-way ANOVA subsequently by Tukey’s HSD post hoc test for multiple comparisons (*** *p* < 0.001, ** *p* < 0.01, * *p* < 0.05). NC: Normal control, STZ: streptozotocin-treated group, STZ + MR: Streptozotocin-treated rats received MR extract, STZ + Mef: Streptozotocin-treated rats received Metformin.

In the case of diabetes-induced kidney injury, the microalbuminuria level is a representative parameter in the urine [17]. We found that STZ-treated rats increased microalbuminuria and serum creatinine concentration in the urine. The concentration of microalbumin excreted in urine and serum creatinine was markedly restored after 14 days of administration of the MR extract (200 mg/kg) or Mef (200 mg/kg) (Figure 2A,B). The urinary volume and urinary creatinine level was also restored after administration of the MR extract to the STZ-treated rats (Supplementary Figure S2A,B). To further confirm the protective effect of MR extract against STZ-induced kidney toxicity, western blotting was performed for urinary excretion of biomarkers (KIM-1, SBP-1, NGAL, and PKM2). The oral administration of the MR extract or Mef reduced the expression of nephrotoxicity biomarkers, which was upregulated markedly in STZ-treated rats (Figure 2C,D). The concentration of the kidney injury marker (3-IS) was also determined [18]. STZ increased 3-IS concentration in the urine, serum, and kidney, and the resulting high 3-IS level was normalized after administration of the MR extract or Mef (Figure 2E).

### 2.7. MR Extract Reduced AGE Levels in STZ-Treated Rats

**Figure 3:**
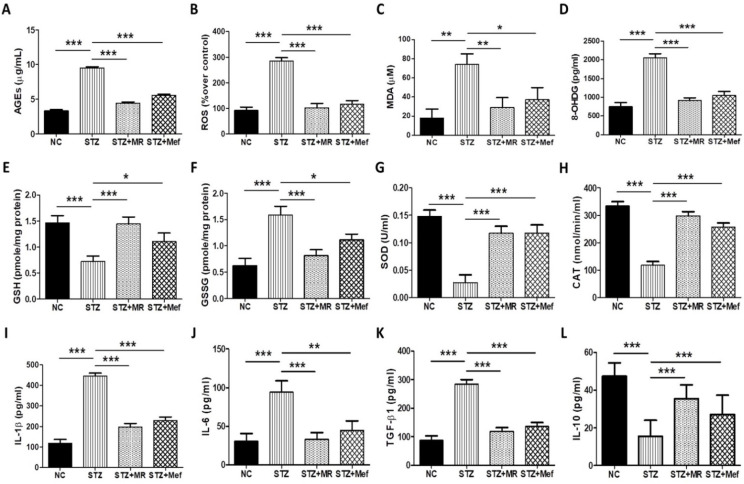
Effects of MR extract on oxidative and inflammatory stress in STZ-treated rats. Levels of (**A**) AGEs, (**B**) intracellular ROS, (**C**) MDA, and (**D**) the oxidative DNA damage marker (8-OHdG) were measured in the kidneys of diabetic animals. (**E**–**H**) Variations in oxidative biomarkers (GSH, GSSG, SOD, and CAT) were evaluated in the kidneys of diabetic animals. Data are expressed as the mean ± S.D. of duplicate experiments (*n* = 6). (**I**–**L**) Changes in the level of the pro-inflammatory cytokines in STZ-treated rats. Data are expressed as the mean ± S.D. of duplicate experiments (*n* = 6). Statistical analysis was completed by one-way ANOVA subsequently by Tukey’s HSD post hoc test for multiple comparisons (*** *p* < 0.001, ** *p* < 0.01, * *p* < 0.05). NC: Normal control, STZ: streptozotocin-treated group, STZ + MR: Streptozotocin-treated rats received MR extract, STZ + Mef: Streptozotocin-treated rats received Metformin.

In hyperglycemia, the extensive development of AGEs is responsible for the progress of diabetic nephropathy [19]. In patients with chronic renal disease, the AGE concentration in serum is not correlated appropriately with diabetic events, possibly because serum concentrations of AGEs are not associated with its deposition in the target tissues. Hence, we measured the AGE amounts in the kidney of STZ-treated rats. STZ treatment drastically increased AGE levels in the kidney but was normalized after administration of the MR extract (200 mg/kg) or Mef (200 mg/kg) (Figure 3A).

### 2.8. Effect of the MR Extract on Oxidative Biomarkers in STZ-Administered Rats

We quantified the concentration of ROS and MDA in the renal tissues of STZ-treated rats, as augmentation of oxidative stress is directly related to elevated AGE concentration in the case of patients with diabetes. After the administration of the MR extract (Figure 3B,C), the levels of ROS and MDA were markedly reduced; these amounts were elevated in STZ-treated rats as compared to the control group. The 8-OHdG (important factor in oxidative DNA damage) concentration was drastically elevated in diabetic-induced rats, which was restored after administration of the MR extract and Mef (Figure 3D). GSH (a non-enzymatic antioxidant) level was reduced markedly in diabetic rats; however, the GSSG level was elevated in diabetic rats compared to the normal control group. GSH and GSSG amounts were restored in STZ-treated rats after administration of the MR extract or Mef (Figure 3E,F). The concentration of SOD (an essential oxidative protein for maintaining mitochondrial functions) was distinctly decreased in STZ-treated rats. However, administration of the MR extract or Mef significantly ameliorated SOD levels (Figure 3G). Furthermore, the concentration of CAT in STZ-administration rats was restored after administration of the MR extract or Mef (Figure 3H).

### 2.9. Protective Effect of the MR Extract on Inflammatory Cytokines in Diabetic Rats

The impact of the MR extract on the maintenance of inflammatory cytokine levels was examined by ELISA. Significant increases in the serum concentrations of inflammatory cytokines (IL-1β, IL-6, and TGF-β1) were observed in diabetic rats, whereas the amount of the anti-inflammatory cytokine IL-10 was reduced (Figure 3I–L). After administration of the MR extracts or Mef, these inflammatory cytokine levels were normalized in diabetes rats (Figure 3I–L).

### 2.10. Protective Effect of the MR Extract on Renal Fibrosis in Diabetic Rats

**Figure 4:**
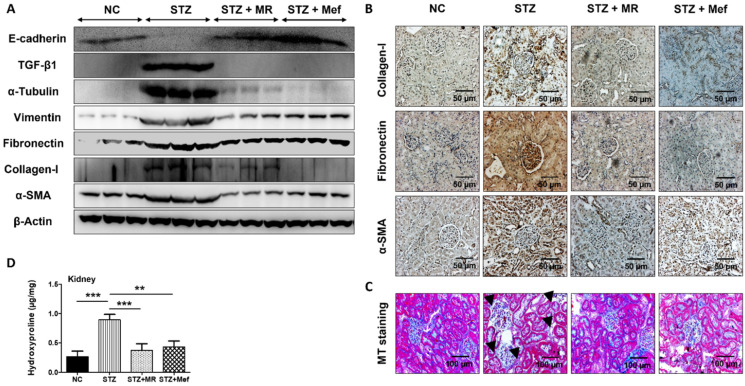
Effects of the MR extract on expression of renal fibrosis biomarkers in STZ-treated rats. (**A**) Expression pattern of E-cadherin, TGF-β1, α-tubulin, vimentin, fibronectin, collagen-1, and α-SMA in the kidney of rats, as determined by western blotting. β-Actin was used as the loading control. The results represent three independent experiments. (**B**) Immunohistochemical analysis of collagen-I, fibronectin, and α-SMA in the kidneys of animals from all groups. Original magnification: 200×, scale bar: 50 μm. (**C**) Images of kidney sections stained with Masson’s trichrome, which indicated renal collagen deposition (blue). Original magnification: 200×, scale bar: 100 μm. (**D**) Level of 4-hydroxyproline content in the serum of STZ-treated rats. Data are expressed as the mean ± S.D. of duplicate experiments (*n* = 6). Statistical analysis was performed by one-way ANOVA followed by Tukey’s HSD post hoc test for multiple comparisons (*** *p* < 0.001, ** *p* < 0.01). NC: Normal control, STZ: streptozotocin-treated group, STZ + MR: Streptozotocin-treated rats received MR extract, STZ + Mef: Streptozotocin-treated rats received Metformin.

The deposition of ECM proteins, which leads to renal fibrosis, increased markedly in glomerular and tubular cells in diabetic-induced hypertrophy. Therefore, the expression of ECM-related proteins was measured in the kidneys of rats. The expression of TGF-β, α-tubulin, vimentin, fibronectin, collagen-1, and α-SMA was upregulated in STZ-induced rats (Figure 4A and Supplementary Figure S3). The administration of the MR extracts or Mef ameliorated the ECM-proteins deposition in the kidney (Figure 4A and Supplementary Figure S3). Furthermore, we confirmed the expression of collagen-1, fibronectin, and α-SMA in the kidney by IHC staining. As shown in Figure 4B, the expressions of these proteins (stained as brownish granules) were higher in STZ-treated rats and were markedly reduced after the administration of the MR extracts and Mef.

In addition, renal fibrosis was investigated by MT staining to determine the degree of collagen deposition in diabetic kidneys. Diabetic rats resulted in the deposition of a high level of collagen in renal tissues followed by nodular formation (Figure 4C), which was reduced after administration of the MR extract or Mef. The hydroxyproline concentration was also drastically increased in the kidney tissues of STZ-treated diabetic rats. However, administration of the MR extracts or Mef reduced hydroxyproline concentration in the kidney to a similar level as in normal control rats (Figure 4D).

### 2.11. Effects of the MR Extract on SIRTs and Claudin-1 Expression in the Renal Cortex of STZ-Treated Rats

**Figure 5:**
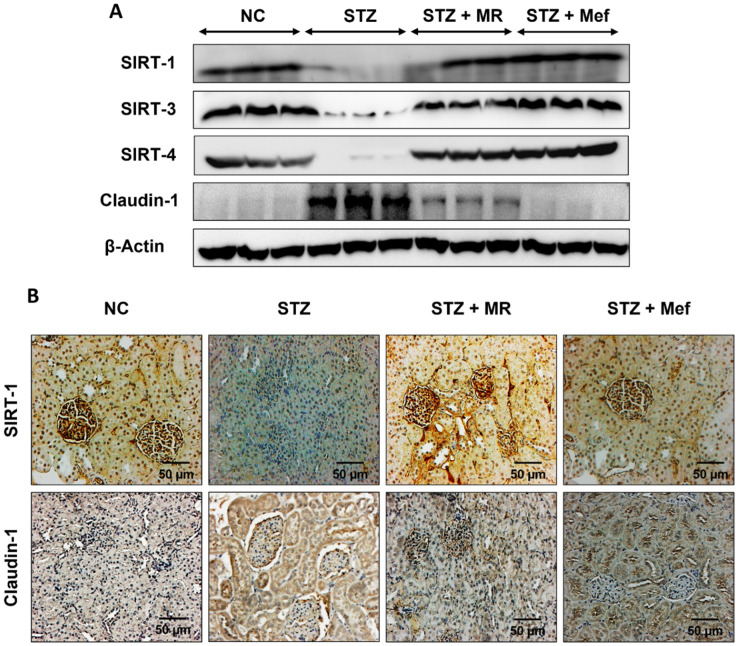
Effects of MR extract on SIRTs and claudin-1 expression in the kidneys of diabetic rats. (**A**) western blotting analysis of SIRT1, SIRT3, SIRT4, and claudin-1 expression. β-Actin expression was used as the loading control. The results represent three independent experiments. (**B**) Immunohistochemical analysis of SIRT-1 and claudin-1 in the kidneys of mice with STZ-induced diabetes. Original magnification: 200×, scale bar: 50 μm. NC: Normal control, STZ: streptozotocin-treated group, STZ + MR: Streptozotocin-treated rats received MR extract, STZ + Mef: Streptozotocin-treated rats received Metformin.

SIRTs and claudin-1 are involved in metabolic disorders resulting from diabetic nephropathy [20,21]. In this study, the SIRT1, SIRT3, and SIRT4 expression was markedly downregulated in the renal cortex of STZ-treated rats compared to the normal control group. However, administration of the MR extracts markedly increased the expression of SIRT1, SIRT3 and SIRT4 in the kidney of STZ-treated rats. Claudin-1 expression was upregulated in STZ-treated rats, but noticeably downregulated after administration of the MR extract or Mef (Figure 5A and Supplementary Figure S4). Furthermore, SIRT1 and claudin-1 expression was also assessed using IHC staining. A robust higher expression of SIRT1 was observed in the kidneys of glomeruli of the normal control group, whereas claudin-1 levels were increased in the renal interstitial tubules of the STZ-treated group. Administration of the MR extracts restored the expression of SIRT1 and claudin-1 in the glomeruli and interstitial tubules (Figure 5B).

## 3. Discussion

Since primordial times, natural medicinal plants containing different phytochemicals such as alkaloids, glycosides, steroids, flavonoids, tannins, and polysaccharides, which help to cure various diseases, have been used [22,23,24,25,26,27,28]. Previous studies have shown that the methanol extracts from the leaves of MR contain several compounds including steroids, alkaloids, and flavonoids. Owing to their pharmacological activities such as antioxidant [29,30] and anti-inflammatory properties [31,32], MR extract has been used traditionally for the treatment of kidney damage. Here, we examined the defensive role of the MR extract against STZ-induced DN.

STZ produces a necrotic effect on pancreatic beta cells and decreases insulin secretion. Hence, it is used widely to establish the type I diabetic model [33,34]. Blood glucose level was markedly increased after 5 days of STZ treatment. Nonetheless, the blood glucose level was restored after 14 days of treatment with the MR extract (200 mg/kg) or Mef (200 mg/kg) administration, which proved that the MR extract restrains the adverse effects of hyperglycemia, thereby aiding in the management of renal abnormalities. Prolonged hyperglycemia caused by STZ treatment reduced the body weight of diabetic rats, which was restored after MR treatment; this showed the protective effect of MR on the muscle tissues damaged by hyperglycemia. Owing to hypertrophy, the weight of the kidney in STZ-treated rats was increased compared to normal rats, but the weight was normalized after the administration of the MR extract or Mef. The administration of the MR extract protected the renal structural and functional parameters, which reflected the protective role of the MR extract against tubular damage by STZ-induced diabetic nephrotoxicity. The MR extract successfully restored all the structural and functional abnormalities including interstitial fibrosis, glomerular collapse, hemorrhage within tubules and glomeruli, and tubular degeneration in STZ-induced diabetic rats [6,35].

The urinary level of microalbumin is a critical marker of diabetic nephropathy [36,37]. STZ treatment resulted in an abnormal increase in microalbumin and creatinine levels, which was suppressed by treatment with the MR extract or Mef. Tubular and glomerular damage was detected by the analysis of urinary protein-based biomarkers [38,39,40,41,42]. The urinary secretion of KIM-1, SBP-1, NGAL, and PKM2 was markedly increased in the STZ-treated group and was normalized after the administration of the MR extract. This reflects that diabetic renal injury develops by complex mechanisms of hyperglycemia, oxidative stress, and inflammation [43,44].

According to Won et al., 2016, 3-IS is a vital renal injury biomarker present in the serum, urine, and kidney tissues, and it was markedly increased in both the serum and renal tissue in diabetes model [18]. In this study, we also observed a significantly elevated level of 3-IS in diabetic rats, which was recovered after MR treatment. The accretion of 3-IS in the kidney was linked to secretion ability and signified tubular damage. Moreover, ROS production in the tubular region was increased and NF-κB was activated at a high concentration of 3-IS, which was directly correlated with plasminogen activator inhibitor-1 [45]. In the case of patients with chronic kidney disease, 3-IS concentration in serum was increased [46,47]. During kidney injury, a high concentration of 3-IS reduces superoxide scavenging activity [48]. Our results showed that the MR extract may have the ability to prevent ROS generation by hindering the accumulation of 3-IS in the kidney of STZ-induced diabetic rats.

Relatively high concentrations of AGEs play an important function in the progression of diabetic nephropathy [19]. In diabetic nephropathy, a high concentration of AGEs is not only associated with the structural abnormalities of renal cortex and blood vessels. Therefore, targeting AGEs can leads to a therapeutic regime against diabetic nephropathy [35,49]. The kidneys are highly prone to the deposition and development of AGEs [50], and elevated accumulation of AGEs can damage the kidneys and their surrounding blood vessels [49]. The concentration of AGEs is directly associated with the degree of abnormality in the kidney structure [35]. During hyperglycemia, AGEs are highly deposited in the kidney, enhancing ROS generation and ultimately injuring the kidney. These make AGEs an attractive therapeutic target for the prevention of diabetic nephropathy. Here, we showed that the MR extract markedly inhibited AGE formation in the kidneys of STZ-treated diabetic rats. This result aligned well with a previous study, where it was found that the overproduction of AGEs increased ROS generation in diabetic rats [51,52]. Thus, the modification of antioxidant enzymes is a possible therapeutic regimen for diabetic nephropathy.

Insulin resistance increases the oxidative stress that simultaneously increased glyco-oxidation and lipid peroxidation [53]. When the concentration of unstable ROS surpasses the cellular defense mechanism, it interacts with vital cellular macromolecules that leads to tissue damage and functional abnormalities [54]. Apart from the biological functional changes, increased level of oxidative stress also reduced serum- antioxidant enzyme activities, increased cellular leakage, and lead to the loss of functional integrity of renal membrane, which in turn increased the glomerular filtration rate and glomerular sclerosis [55,56]. Antioxidants neutralize the harmful free radicals and neutralize the oxidative cellular damage. Physiological changes in concentration can effect on the resistance of cellular DNA, proteins and lipids to oxidative damage [55,56]. In presence of high glucose concentration, MDA level is increased in vascular smooth muscle cells, proximal tubule cells, and mesangial cells. In diabetic patients, a high concentration of MDA is found in the renal cortex, plasma, and aorta [57]. Hence, antioxidant controlling opens up a new therapeutic strategy for the patients with diabetic nephropathy.

In hyperglycemia-induced diabetic nephropathy, a high level of blood glucose increased ROS generation, producing a high level of oxidative stress, which causes renal failure [58,59,60,61]. In this study, the decrease in antioxidant potential due to hyperglycemia in STZ-induced diabetic rats was improved by the administration of the MR extract, with the marked restoration of the SOD and CAT levels. MDA and 8-OHdG levels were increased in the kidneys of STZ-treated rats, and the administration of the MR extract markedly reduced MDA and 8-OHdG. In diabetic nephritic disorders, 8-OHdG concentration is increased in the urine [50,62]. Hyperglycemia induces oxidative stress; hence, the GSH to GSSG ratio was decreased [63]. The concentration of GSH and GSSH was higher and lower, respectively, in the STZ-treated diabetic rats than in the normal control group, and the administration of MR restored this abnormality.

Cytokines play an important role in intercellular kidney injury and produce secondary messengers such as acute phase proteins, cell adhesion molecules, and transcription factors [64]. According to clinical data, the concentration of IL-1β and IL-6 was increased in patients with diabetic nephropathy [65]. In our study, STZ-treatment increased the concentrations of both IL-1β and IL-6. Although the exact molecular mechanism through which pro-inflammatory cytokines affect the regulation of diabetic nephropathy is not determined, it has been theorized that pro-inflammatory cytokines are closely associated with the thickening of the basement membrane and podocyte abnormalities [66]. According to Shahzad et al., 2015, IL-1β levels were elevated in the renal cortex and the serum of db/db mice in an age-dependent manner [67]. Here, the administration of the MR extract markedly reversed the increase in IL-1β levels. Our study supported other studies, which also reported that the IL-6 concentration was unusually increased in STZ-induced diabetic rats and was normalized after administration of the MR extract. Owing to the high concentration of IL-6, thickening of the glomerular membrane, mesangial cell proliferation, and alteration of endothelial permeability were detected in DN [68].

Renal fibrosis depends on the accumulation rate of ECM proteins, mesangial cell proliferation, and thickening of the glomerular basement membrane [69,70]. TGF-β1 has a critical role in the maintenance of diabetic-induced renal fibrosis, which is regulated by the α-SMA expression [71]. In this study, the expression of TGF-β, α-tubulin, vimentin, fibronectin, collagen-1, and α-SMA was upregulated markedly, but E-cadherin expression was downregulated after STZ treatment and was further decreased by MR administration. These data were further supported by the IHC staining for collagen-1, fibronectin, and α-SMA; STZ increased their expression level, which was restored by treatment with the MR extract. The administration of the MR extract decreased collagen deposition, which was confirmed by MT staining. Hydroxyproline is a well-known basic marker of kidney fibrosis [42]. Its levels were considerably restored after MR treatment in STZ-induced diabetic rats. Therefore, the MR extract possesses anti-fibrotic activity against diabetic-induced renal injury.

SIRT1 plays a vital role in the metabolism and inflammation of diabetic-induced renal injury [20,72] and reduced activity of SIRT1 in the renal cortex is common in diabetic disorders [21]. Hence, it is a possible target in the treatment of DN [73,74]. The expression of SIRT1 and claudin-1 were negatively correlated with each other both in the glomerular sections and proximal tubules [75]. An increase in SIRT1 expression level restored diabetic renal injury and impaired ROS-mediated apoptosis in mesangial cells [76,77]. In our study, the administration of the MR extracts upregulated SIRT1 and downregulated claudin-1 protein expression in the STZ-induced group. SIRT3 inhibits the inflammation associated with oxidative stress [78] and can scavenge ROS to protect kidney tissues against cell senescence and apoptosis [79,80]. It is an essential constituent of the acetyl-proteome in the mitochondria [81], and the upregulated expression of SIRT3 improved mitochondrial dysfunction and protected against renal injury. In this study, the MR extract upregulated SIRT3 expression in diabetic rats. SIRT4 also plays a role in the regulation of mitochondrial function and the pathogenesis of metabolic disorders including renal dysfunction, but its exact role in diabetic-induced kidney damage remains unclear. In the mitochondria, SIRT4 assists in the conversion of glutamate to α-ketoglutarate and controls insulin secretion from pancreatic β-cells in response to glucose and amino acids [82]. Our western blotting results showed that STZ-treatment markedly downregulated SIRT4 expression, which was consistent with the results of previous studies in a mouse model of type-2 diabetes [83] and insulin-resistant rats [84]. Our immunohistochemistry results also supported the western blotting data. The overexpression of SIRT4 improved renal injury in hyperglycemic conditions.

The pharmacological effect of our crude leaf extract on blood glucose level and it’s possible mechanism depend on the modulation of sirtuin proteins (SIRT1, SIRT3, and SIRT4). SIRT1 possesses anti-inflammatory activity via deacetylation or inactivation of the p65 subunit by reducing ROS production. It also regulates apoptosis, metabolism, and mitochondrial biogenesis [85,86,87]. The downregulation of SIRT1 in proximal tubule upregulates the claudin-1 expression in podocyte that results in the interruption of glomerular filtration, podocyte malfunction, and albuminuria [75]. Our current study also revealed the lower expression of SIRT1 in DN model. However, after treatment with the MR extract, the expression of SIRT1 was restored. The anti-inflammatory role of SIRT3 is associated with oxidative stress in diabetes induced kidney injury. This protective function of SIRT3 is highly related to the reduction in ROS production and attenuation of inflammation formation [78,79,80]. SIRT4 overexpression inhibits apoptosis and increases proliferation along with reduced ROS production and increased mitochondrial membrane potential. Under hyperglycemic conditions, SIRT4 overexpression may decrease podocyte injury and inhibit podocyte apoptosis [88]. Therefore, the up regulation of SIRT1, SIRT3 and SIRT4 and downregulation of Claudin 1 by MR extract might prevent renal fibrosis by suppressing mitochondrial oxidative stress and the production of inflammatory cytokines. However, further experiments are necessary to identify the individual isolated compound and its mechanisms in our study.

In our study, the administration of the MR extract upregulated the expression of all SIRT proteins (SIRT-1, SIRT-3, and SIRT-4), which may be associated with the protective effect of the MR extract against DN. Our data suggested that the MR extract has an excellent therapeutic efficacy against DN. However, our extract has the potential to reduce the STZ-induced diabetic nephropathy, but isolation and characterization of the crude extract limits our present study. Further research is necessary to characterize the active constituent behind this protective effect of the MR extract against STZ-induced diabetic nephropathy to unfold the mechanistic approach.

## 4. Materials and Methods

### 4.1. Chemicals and Reagents

Streptozotocin (STZ) and metformin (Mef) were acquired from Sigma–Aldrich Biotechnology (St. Louis, MO, USA). The primary antibodies including kidney injury molecule-1 (KIM-1), neutrophil gelatinase-associated lipocalin (NGAL), selenium binding protein-1 (SBP1), pyruvate kinase muscle isozyme M2 (PKM2), collagen-1, E-cadherin, TGF-β1, vimentin, fibronectin, α-tubulin, claudin-1, α-SMA, SIRT1, SIRT3, SIRT4, and β-actin were obtained from Abcam (Cambridge, MA, USA). Immobilon Forte Western HRP substrate (cat. no. WBLUF0100) and polyvinylidene difluoride (PVDF) membrane were procured from Millipore (Burlington, MA, USA). Other chemicals were obtained from Sigma–Aldrich (St. Louis, MO, USA).

### 4.2. Preparation of the MR Extract

MR leaves were collected in February and March from the rural belt of Tripura (India). This plant was authenticated by the Dr. B. K. Datta (Department of Botany, Tripura University, India). The reference number was BOT/HEB/AC7228. The leaves were cleaned with distilled water, dried in the shade, powdered, and then extracted with methanol (1 L) at 90 °C for 6 h. The solvent was removed under reduced pressure in a rotary evaporator and MR extracts were stored in a sealed container at 4 °C until required [14,15].

### 4.3. Design of Animals Experiment and Acute Toxicity Study

Eight-week-old Sprague-Dawley male rats (body weight 250 ± 5 g) were purchased from Charles River Animal Laboratories (Orient, Seoul, Korea) and accommodated in a specific-pathogen-free (SPF) room with a 12 h light/dark cycle. The relative air temperature and humidity were set at 23 ± 0.5 °C and 55 ± 2%, respectively. Before the start of the experiment, the animals received a 2-week adjustment period. All rats were given ad libitum access to tap water and food (PMI, Brentwood, MO, USA). This study was approved by Sungkyunkwan University Laboratory Animal Care Service (A20180420-2865) following the regulations of the Korea Food and Drug Safety Administration (KFDA). The oral toxicity study of the MR extract was performed following OECD guidelines. MR extract (500 and 1000 mg/kg) was administered orally to Sprague-Dawley rats for 7 days, and the rats were observed for toxicity symptoms including diarrhea, changes in sleep, changes in skin color, convulsions, and respiratory symptoms.

### 4.4. Experimental Design

**Figure 6:**
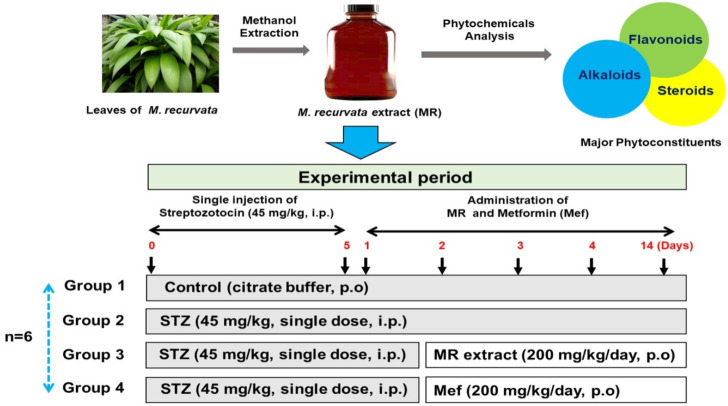
Experimental plan. Diabetes was induced by a single intraperitoneal (i.p.) injection of STZ (45 mg/kg). Citrate buffer was administered to the normal control group (*n* = 6). After 5 days of STZ treatment, the remaining rats were divided randomly into three groups: the STZ group (*n* = 6), STZ + MR (200 mg/kg of MR extract) group (*n* = 6), and the STZ + Mef (200 mg/kg) group (*n* = 6). The subsequent two groups (group 3 and 4) received MR extract and Mef (standard drug), respectively, for a total of 14 days. STZ: Streptozotocin, Mef: Metformin, and MR: *Molineria recurvate*. p.o.: mouth orally.

In all rats, diabetes was induced by a single intraperitoneal (i.p.) injection of STZ (45 mg/kg; dissolved in 0.1 M cold citrate buffer, pH 4.5). After 5 days, the fasting blood glucose level was measured, and the animals with blood glucose levels higher than 300 mg/dL were considered diabetic and selected for subsequent experiments. The experimental design is shown in Figure 6. The rats were divided into four groups (*n* = 6 animals per group): Group 1, the normal control group (NC), in which the animals received citrate buffer by oral gavage for 14 days; Group 2, the STZ group (STZ), in which the animals received a single STZ (45 mg/kg) injection i.p. [6]; Group 3, the STZ + Mef group (standard; STD), in which the animals received STZ injection and Mef (200 mg/kg) daily by oral gavage for 14 days; and Group 4, the STZ + MR extract group (test), in which the animals received STZ injection and MR extract (200 mg/kg) daily by oral gavage for 14 days. According to Figure 1, the MR extract and Mef were administered on day 6 after STZ injection. The Mef dose was selected as 200 mg/kg, based on the study by Niture et al., 2014 [89], which reported the dose to be effective at controlling the glucose level. Moreover, a dose of ≥ 600 mg/kg/day can result in toxic signs and symptoms [90]. Therefore, 200 mg/kg Mef selected for this study was appropriate for regulating the blood glucose level without producing any toxicity. All animals were observed for any clinical abnormality and dose-associated toxicities, and body weight was regularly measured. Blood from the animals was collected from the tail vein, and blood glucose level was measured on days 0, 7, and 14 days using a glucometer (ACCU-CHEK; Daeil Pharm. Co Ltd., Seoul, Korea). Urine samples were collected on days 7 and 14 using a metabolic cage. After sacrifice, major organs (kidney, liver, and pancreas) were perfused with saline to eliminate any trace of blood and then stored at −80 °C for biochemical analysis. Urine and serum samples were frozen at −20 °C for urinary and biochemical analysis.

### 4.5. Histological Analysis

The right kidney of each animal was fixed in formaldehyde with 10% phosphate-buffered saline (PBS). The tissue sections (5-μm slices) were embedded in paraffin and were stained with hematoxylin and eosin (H&E) for studying histopathological alterations. All paraffin sections were de-paraffinized with xylene, rehydrated in a graded alcohol series, rinsed with deionized water, and stained with H&E for 1 min. After washing in tap water for 5 min, the stain was developed. The tissue sections were destained with acidified ethanol, washed in tap water, dehydrated, mounted, and stained with eosin for 30 s. Finally, the stained slides were examined for any histopathological changes using a Zeiss Axiphot light microscope (Zeiss, Carl Zeiss, Oberkochen, Germany).

### 4.6. Urine Analysis and Quantification of Biochemical Parameters

All the animals were shifted to metabolic cages overnight for 24 h before they were sacrificed on day 10. The total urine volume was collected and measured and then centrifuged at 879× *g* and 4 °C for 10 min. The supernatant was collected for the investigation of urinary biomarkers. Furthermore, the blood from each rat was collected from the hepatic vein and centrifuged at 2000× *g* and 4 °C for 10 min to separate the serum. The urine and serum of each rat were kept at −80 °C for subsequent experiments. The quantitative analysis of creatinine, microalbumin, and total protein (from the collected urine samples) was performed using the TBA-200FR NEO urine chemistry analyzer (Toshiba, Tochigi-Ken, Japan).

### 4.7. Detection of 3-Indoxyl Sulfate

The concentration of 3-indoxyl sulfate (3-IS) was quantified in the urine, plasma, and kidney tissue using high-performance liquid chromatography (HPLC), as described in our previous study [88]. The 3-IS was detected and quantified using a C18 column (5 μm; 250 mm × 4.6 mm) from Azilant (Torrance, CA, USA) at a flow rate of 0.9 mL/min at room temperature. First, kidney tissue (100 mg) was homogenized with phosphate buffer, centrifuged, and the supernatant was collected. The plasma, urine, and kidney samples were then extracted with acetonitrile (1:1 ratio; precipitation of protein occurs) and centrifuged (1000× *g*). The resulting supernatants were analyzed for 3-IS. A blank urine sample containing an internal standard (2-naphthalene sulfonic acid) and 10 different concentrations of 3-IS (1.56–800 μg/mL) was used to construct the calibration plots. After extraction, the collected samples were transferred into a sample tube and thoroughly mixed with acetonitrile containing an internal standard (diclofenac sodium salt, Tokyo Chemical Industry, Tokyo, Japan). The isocratic mobile phase (a mixture of 0.2% TFA in Milli-Q water and acetonitrile in a ratio of 80:20 *v*/*v*) was used at a flow rate of 1 mL/min at 25 °C. The wavelength of the IS was 280 nm, and the retention time was 7.5 min.

### 4.8. Examination of Advanced Glycation End Products

AGE assay was completed as per manufacturer’s instructions, as previously described by Kaur et al. [91]. In this ELISA, the AGE samples (test and standard) were blocked in a microplate precoated with the AGE-specific antibody. The wells were washed, incubated, washed again to remove unbound conjugate, and incubated at 37 °C for 1 h. All the wells were again supplemented with an HRP-conjugated secondary antibody and further incubated at room temperature for 1 h. Subsequently, the substrate solution was added and incubated for 2–20 min to develop the color. The absorbance at 450 nm was recorded using a microplate reader.

### 4.9. Evaluation of Oxidative Stress

The kidney tissues (200 mg) from each rat were homogenized in ice-cold HEPES buffer (20 mM, pH 7.2), and then centrifuged (1500× *g*). The protein concentration of the supernatant was quantified using a Protein Assay Kit (500–0006, Bio-Rad, Hercules, CA, USA). Intercellular reactive oxygen species (ROS; Cell Biolabs, San Diego, CA, USA), malondialdehyde (MDA; MDA-586, OxisResearCh™, Portland, OR, USA), reduced forms of glutathione (GSH) and oxidized GSH (GSSG; Cayman Chemical Co., Ann Arbor, MI, USA), superoxide dismutase (SOD) activity, and catalase (CAT) peroxidation (Cayman Chemical Co., Ann Arbor, MI, USA) were quantified in accordance with the manufacturer’s protocol. In contrast, 8-hydroxy-2′-deoxy-guanosine (8-OHdG) in urine samples was investigated using an ELISA assay kit (Cell Biolabs, San Diego, CA, USA) in accordance with the company’s instructions.

### 4.10. Assessment of Inflammatory Cytokines

The inflammatory biomarkers, namely IL-1β, IL-6, IL-10, and TGF-β, of diabetic nephropathy, were investigated using ELISA Kit (Abcam, Cambridge, MA, USA) according to the manufacturer’s protocol.

### 4.11. Investigation of Hydroxyproline

Hydroxyproline was quantified using a hydroxyproline assay kit (Cell BioLabs, San Diego, CA, USA) in accordance with the manufacturer’s protocol, and the levels were reported as micromoles per milligram of protein.

### 4.12. Western Blot Analyses

Western blotting was performed as previously described [92,93,94]. For the urine samples, the urine was centrifuged (1000× *g* for 10 min), and the supernatant (considered to contain the total urinary proteins) was collected and diluted in a 1:2 ratio with double distilled water (DDW). Protein from the kidney tissue was collected using a PRO-PREP cell lysis buffer. The samples were electrophoresed on a 6–12% sodium dodecyl sulfate (SDS) polyacrylamide gel. After electrophoresis, the resolved protein bands were transferred onto PVDF membranes, blocked, incubated with primary and corresponding secondary antibodies, and developed using Immobilon Forte Western HRP substrate. All uncropped western blot images are mentioned in Supplementary Figure S5A–Q.

### 4.13. Immunohistochemical Evaluation

Immunohistochemical (IHC) analysis of rat kidney tissues was performed as previously described [95]. To examine the expression of α-SMA, fibronectin, collagen-1, SIRT1, and claudin-1 in tissues from all groups, the slides were exposed to the appropriate primary antibodies after de-paraffinization, hydration, and antigen retrieval. The slides were moved to a xylene chamber, dipped in alcohol and water, and then incubated with 3% hydrogen peroxide. Subsequently, non-specific binding to the slides was blocked, and the slides were incubated with primary antibodies and then with horseradish peroxidase (HRP)-conjugated secondary antibodies, in accordance with the manufacturer’s instructions. Finally, all immunostained slides were visualized using diaminobenzidine tetrahydrochloride (DAB), counterstained with hematoxylin, and examined using a microscope.

### 4.14. Statistical Analysis

Data are presented as the mean ± standard deviation (S.D.; *n* = 6). One-way analysis of variance (ANOVA) was followed by Tukey’s (honest significant difference) post hoc test for multiple comparisons. Analyses were performed using GraphPad Prism 5 software (Version 5.0, San Diego, CA, USA). Post hoc testing was performed for intergroup comparisons using the least significant difference test. The following levels of statistical significance were considered: *** *p* < 0.001, ** *p* < 0.01, and * *p* < 0.05.

## 5. Conclusions

In our study, methanolic extract of MR leaves markedly normalized STZ-induced increases in the levels of blood glucose, AGEs, oxidative stress, inflammatory cytokines, and kidney function biomarkers, and repaired the histopathological damage to the kidney and pancreas. The administration of the MR extract enhanced the antioxidant capability by increasing GSH, SOD, and CAT levels in the STZ-treated diabetic group. Hence, the MR extract can be utilized as a novel therapeutic agent for renal disorders including diabetic nephropathy. Future experiments will highlight the targets and molecular mechanism of the MR extract to aid the discovery and development of new drugs for diabetic nephropathy.

## Figures and Tables

**Table 1:** Photochemical properties of the MR extracts from the leaf of *Molineria recurvata*.

Phytochemicals	Methanolic Extract
Alkaloid	+++
Carbohydrate	+
Saponin	−
Steroid	++
Sulphate	−
Tannin	−
Flavonoid	++
Hydroxy-anthraquinone glycoside	+
Starch	−
Dextrin	+

+++: Copiously present; ++: moderately present; +: slightly present; −: absent.

## Data Availability

Not applicable.

## Supplementary Materials

The following supporting information can be downloaded at: https://www.mdpi.com/article/10.3390/molecules27154985/s1, Figure S1: Effect of MR extract on the (A) oral glucose tolerance and (B) insulin tolerance in STZ treated diabetic rats. Figure S2: Effect of MR extract on the (A) urinary volume and (B) urinary creatinine level in STZ treated diabetic rats. Figure S3: Effects of MR extract on expression of renal fibrosis biomarkers in STZ-treated rats. The band intensity was analyzed densitometrically using ImageJ software. (*** *p* < 0.001, ** *p* < 0.01, * *p* < 0.05). Figure S4: Effects of MR extract on SIRTs and claudin-1 expression in the kidneys of diabetic rats. The band intensity was analyzed densitometrically using ImageJ software. (*** *p* < 0.001, ** *p* < 0.01, * *p* < 0.05). Figure S5: Uncrop images of the western blots. (A) Kim-1, (B) SBP-1, (C) NGAL, (D) PKM2, (E) E-Cadherin, (F) TGF-b1, (G) 1-Tubulin, (H) Vimentin, (I) Fibronectin, (J) Collagen-I, (K) a-SMA, (L) b-actin, (M) SIRT1, (N) SIRT3, (O) SIRT4, (P) Claudin-1, (Q) b-actin.

## Abbreviations

AGEs	Advanced glycation end products
CAT	Catalase
DAB	Diaminobenzidine tetrahydrochloride
DN	Diabetic nephropathy
ECM	Extracellular matrix
EMT	Epithelial-mesenchymal transition
ESRD	End-stage renal disease
GSH	Glutathione
GPx	Glutathione peroxidase
H&E	Hematoxylin and eosin
HPLC	High-performance liquid chromatography
HRP	Horseradish peroxidase
3-IS	3-indoxyl sulfate
KIM-1	Kidney injury molecule-1
IL-1β	Interleukin-1β
MDA	Malondialdehyde
MR	Molineria recurvata
ROS	Reactive oxygen species
8-OHdG	8-Hydroxy-deoxyguanosine
PBS	Phospahte-buffered saline
PKM2	Pyruvate kinase M2
PVDF	Polyvinylidene difluoride
SBP-1	Selenium binding protein 1
SDS	Sodium dodecyl sulfate
SOD	Superoxide dismutase
STZ	Streptozotocin
α-SMA	α-Smooth muscle actin
TBA	Thiobarbituric acid
TGF-β	Transforming growth factor-β
TNF-α	Tumor necrosis factor-α
