# Supplementary figures and images for: Molineria recurvata Ameliorates Streptozotocin-Induced Diabetic Nephropathy through Antioxidant and Anti-Inflammatory Pathways
Source: Molecules. 2022 Aug 5;27(15):4985. doi: 10.3390/molecules27154985 (PMC9370403; doi:10.3390/molecules27154985)

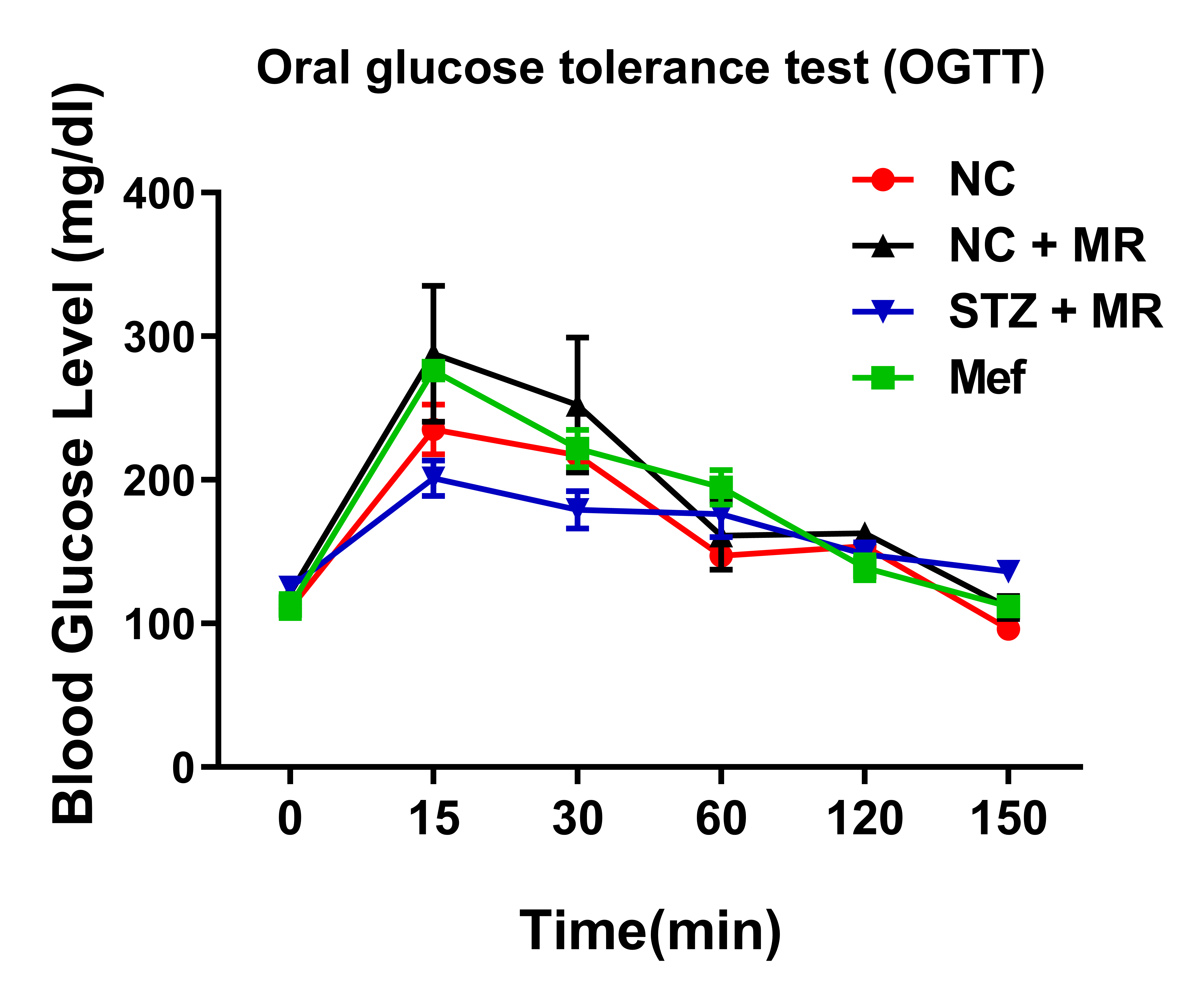

Supplement: Supplementary file 1 [file molecules-27-04985-s001.zip › S1A_Oral glucose tolerance test.tif]

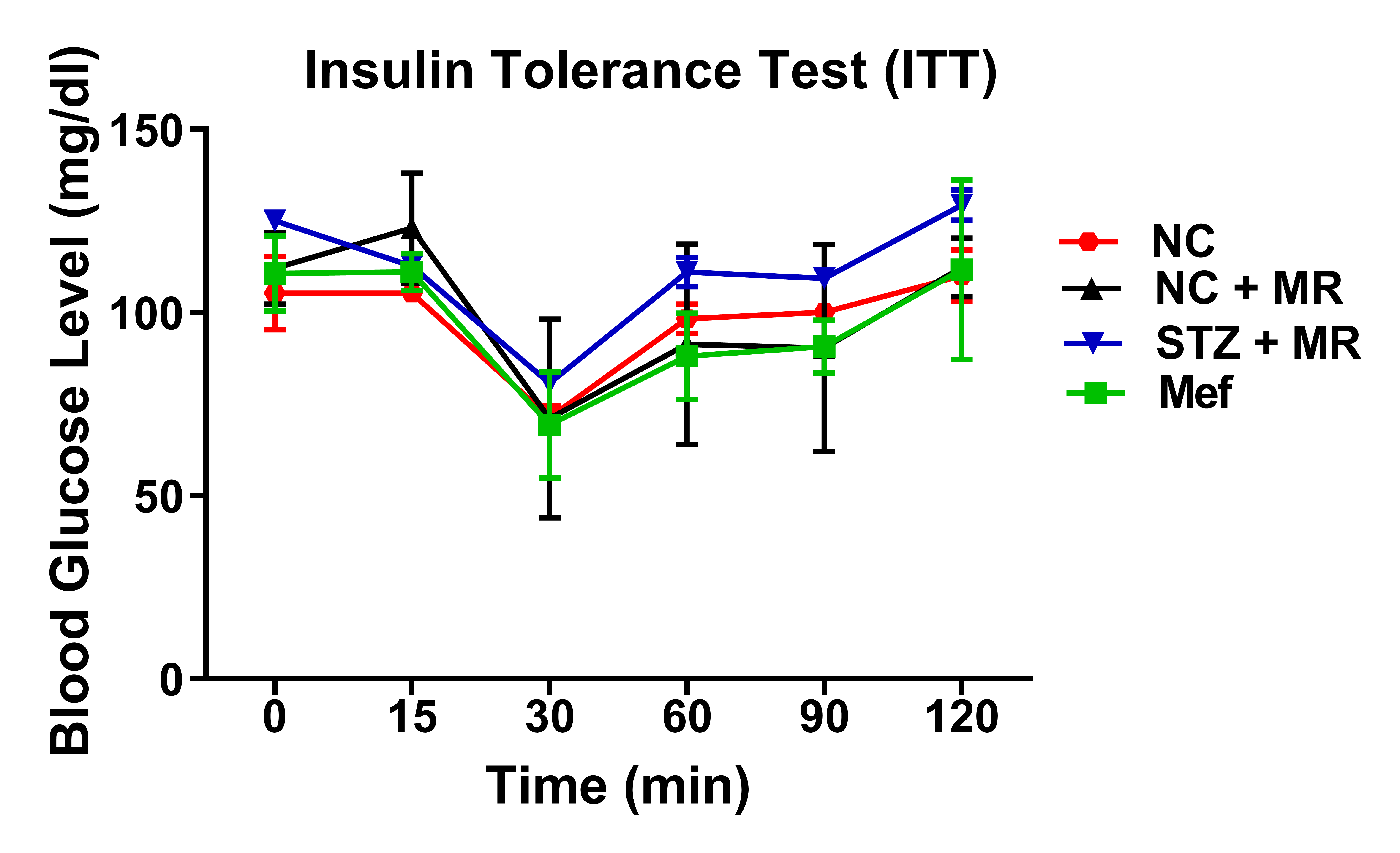

Supplement: Supplementary file 1 [file molecules-27-04985-s001.zip › S1B_Insulin tolerance test.tif]

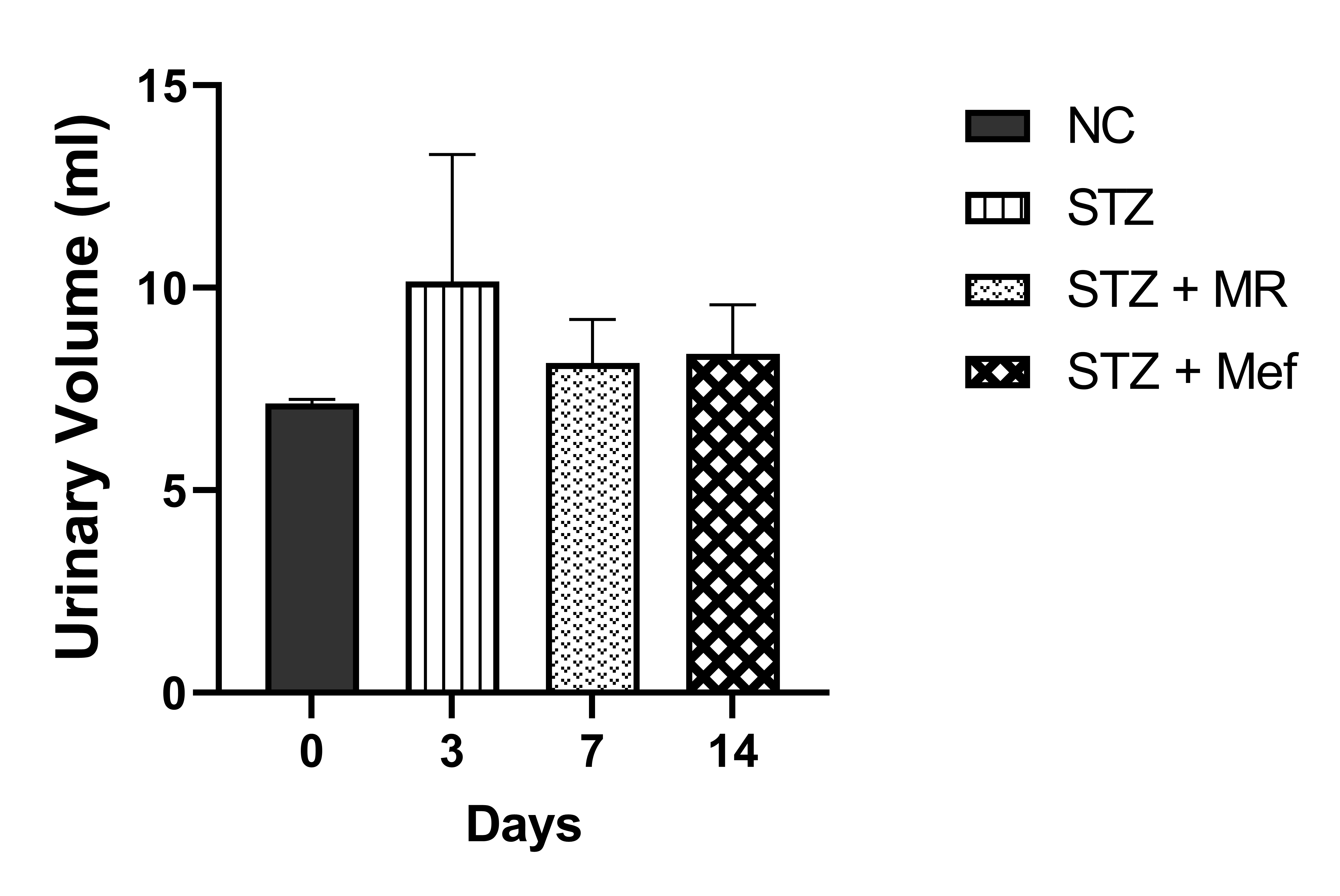

Supplement: Supplementary file 1 [file molecules-27-04985-s001.zip › S2A_Urinary volume.tif]

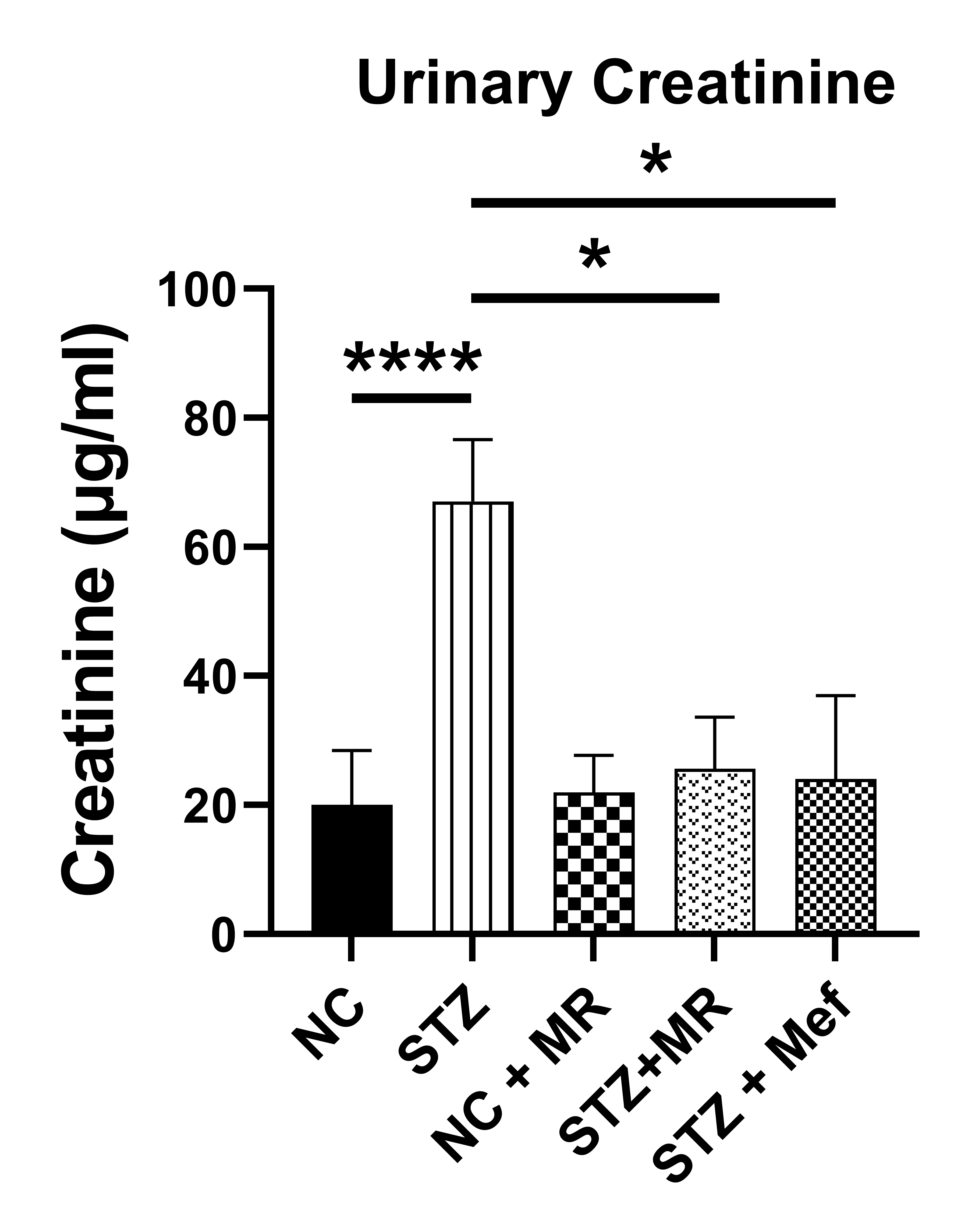

Supplement: Supplementary file 1 [file molecules-27-04985-s001.zip › S2B_Urinary creatinine.tif]

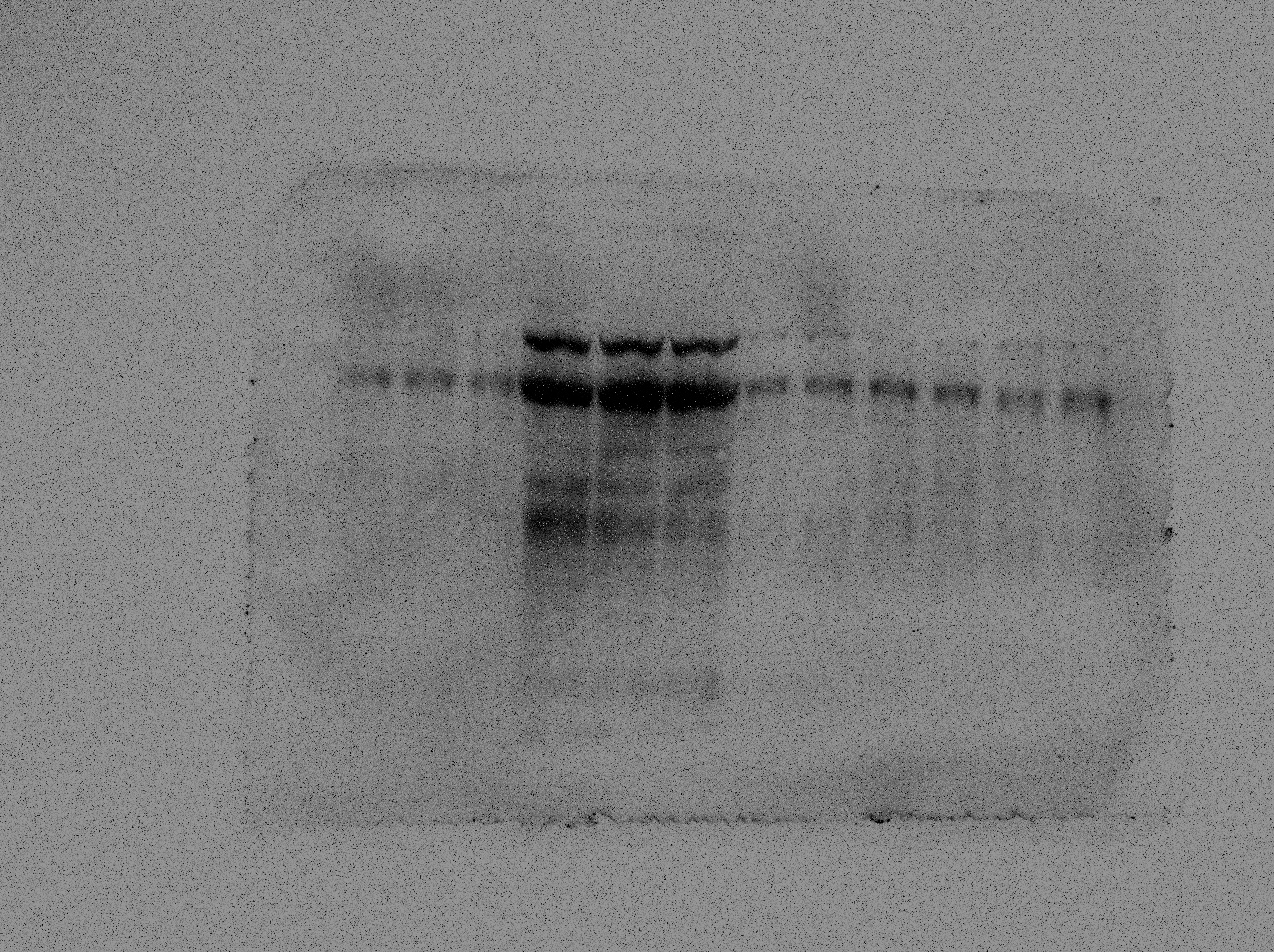

Supplement: Supplementary file 1 [file molecules-27-04985-s001.zip › S5A_Kim-1.jpg]

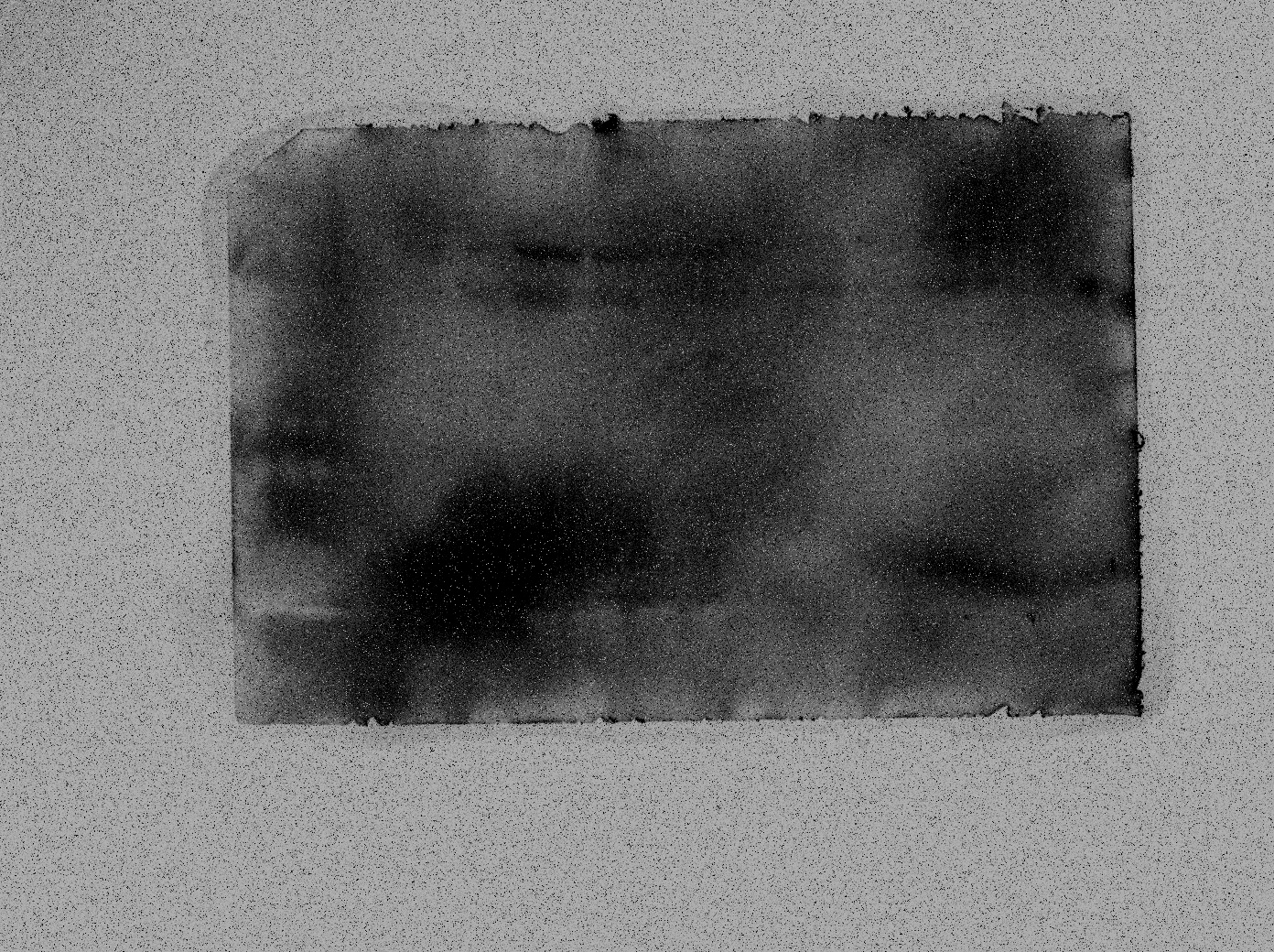

Supplement: Supplementary file 1 [file molecules-27-04985-s001.zip › S5B_SBP-1.jpg]

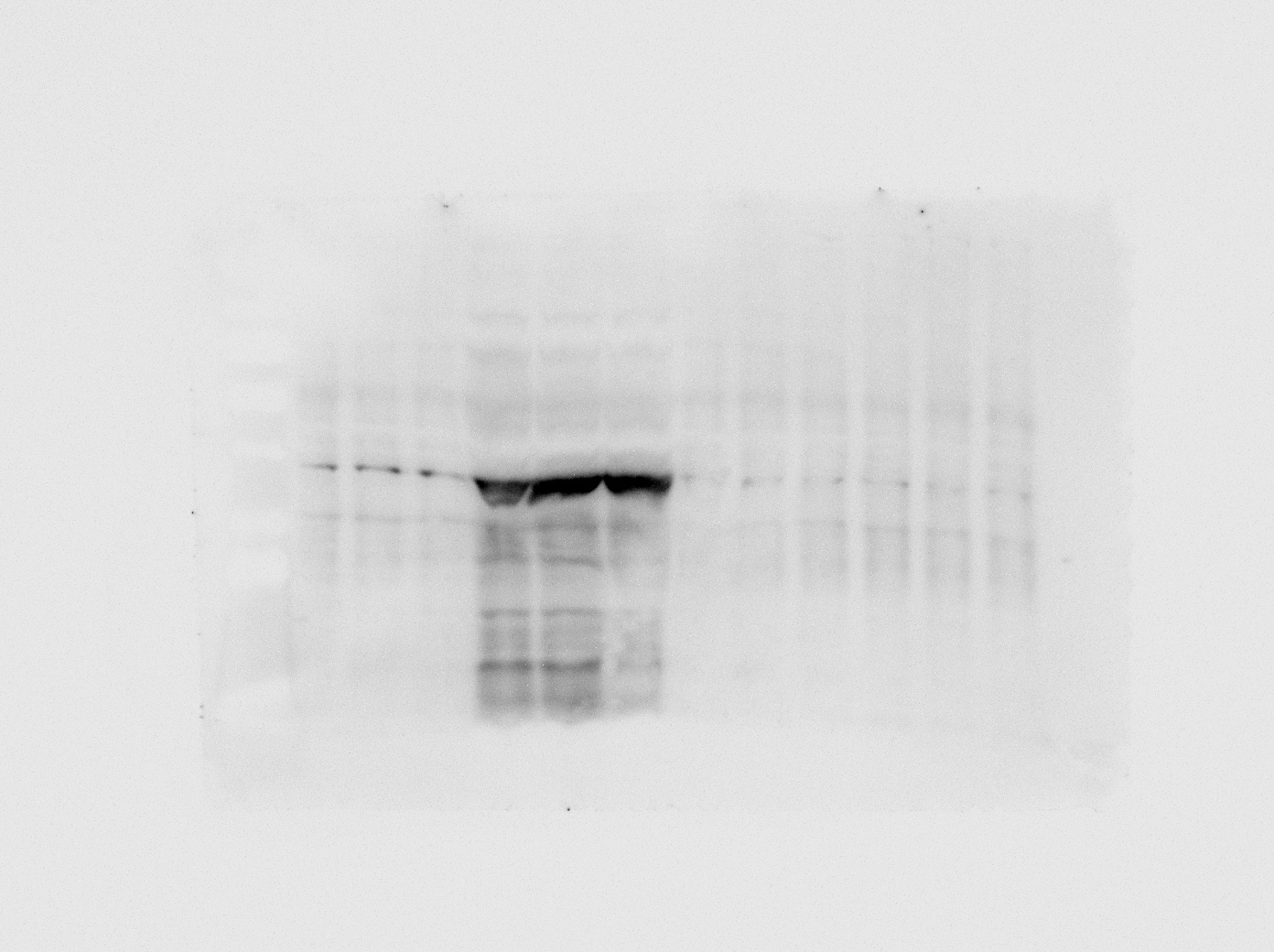

Supplement: Supplementary file 1 [file molecules-27-04985-s001.zip › S5C_NGAL.jpg]

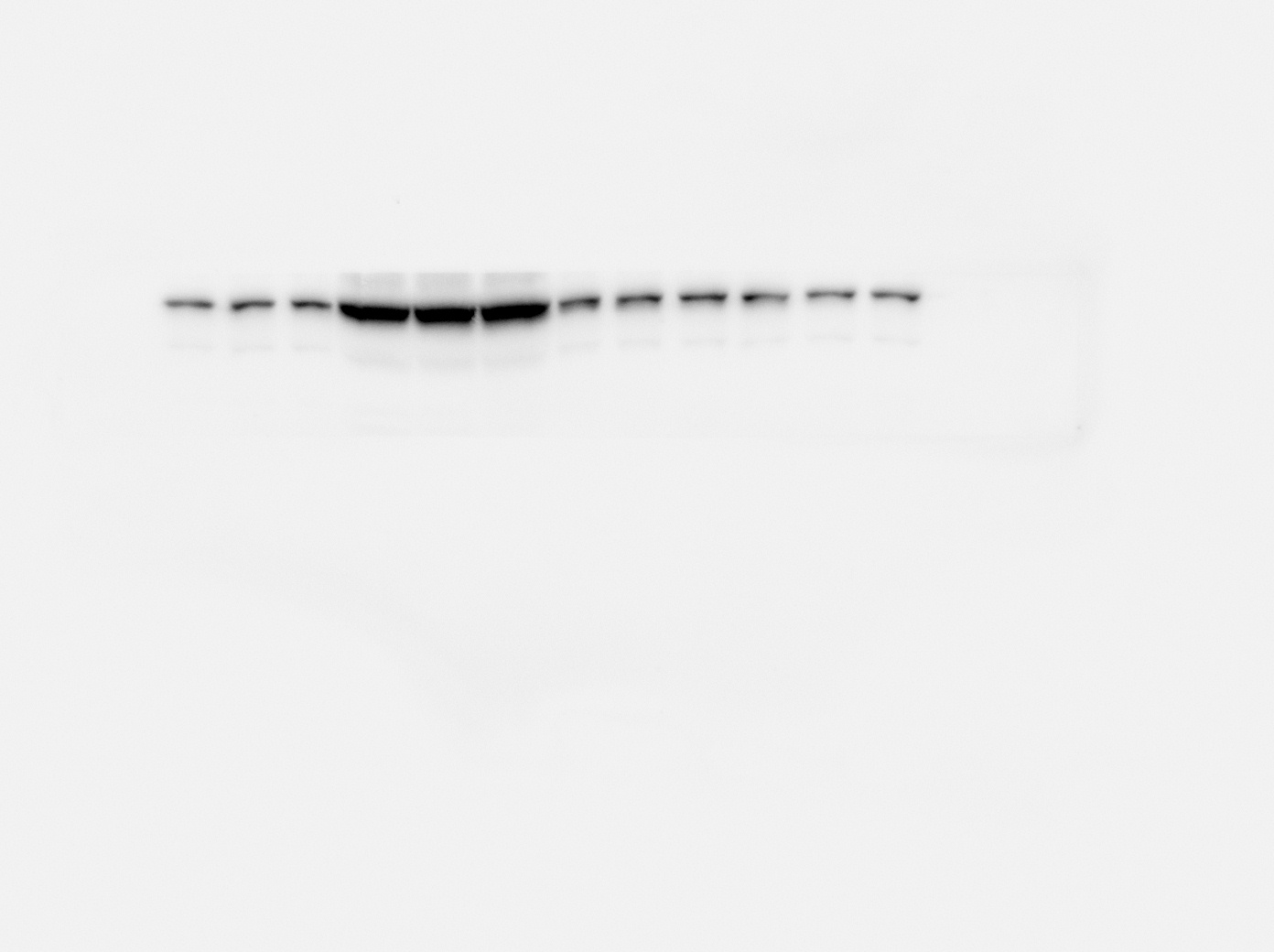

Supplement: Supplementary file 1 [file molecules-27-04985-s001.zip › S5D_PKM2.jpg]

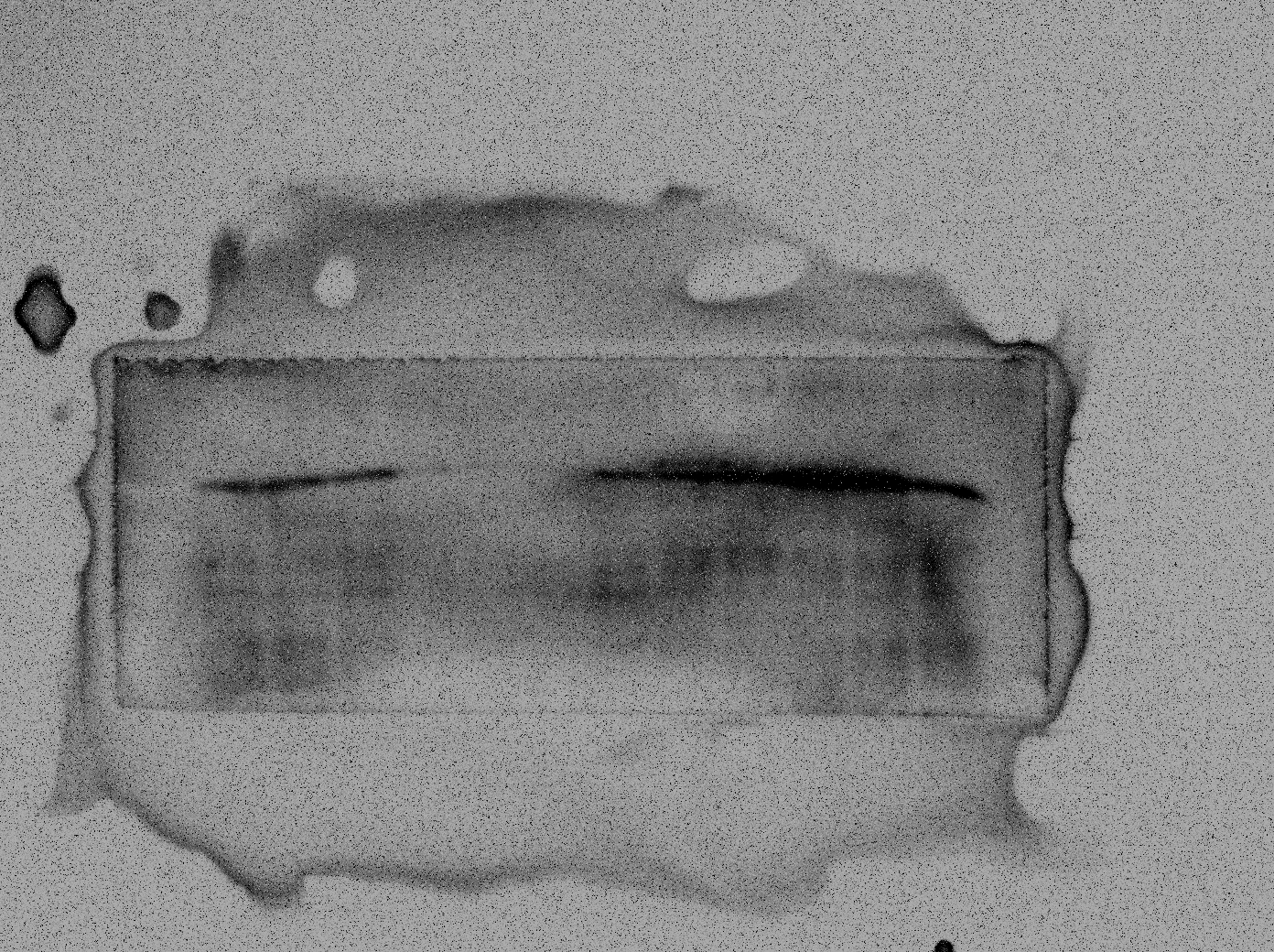

Supplement: Supplementary file 1 [file molecules-27-04985-s001.zip › S5E_E-cadherin.tif]

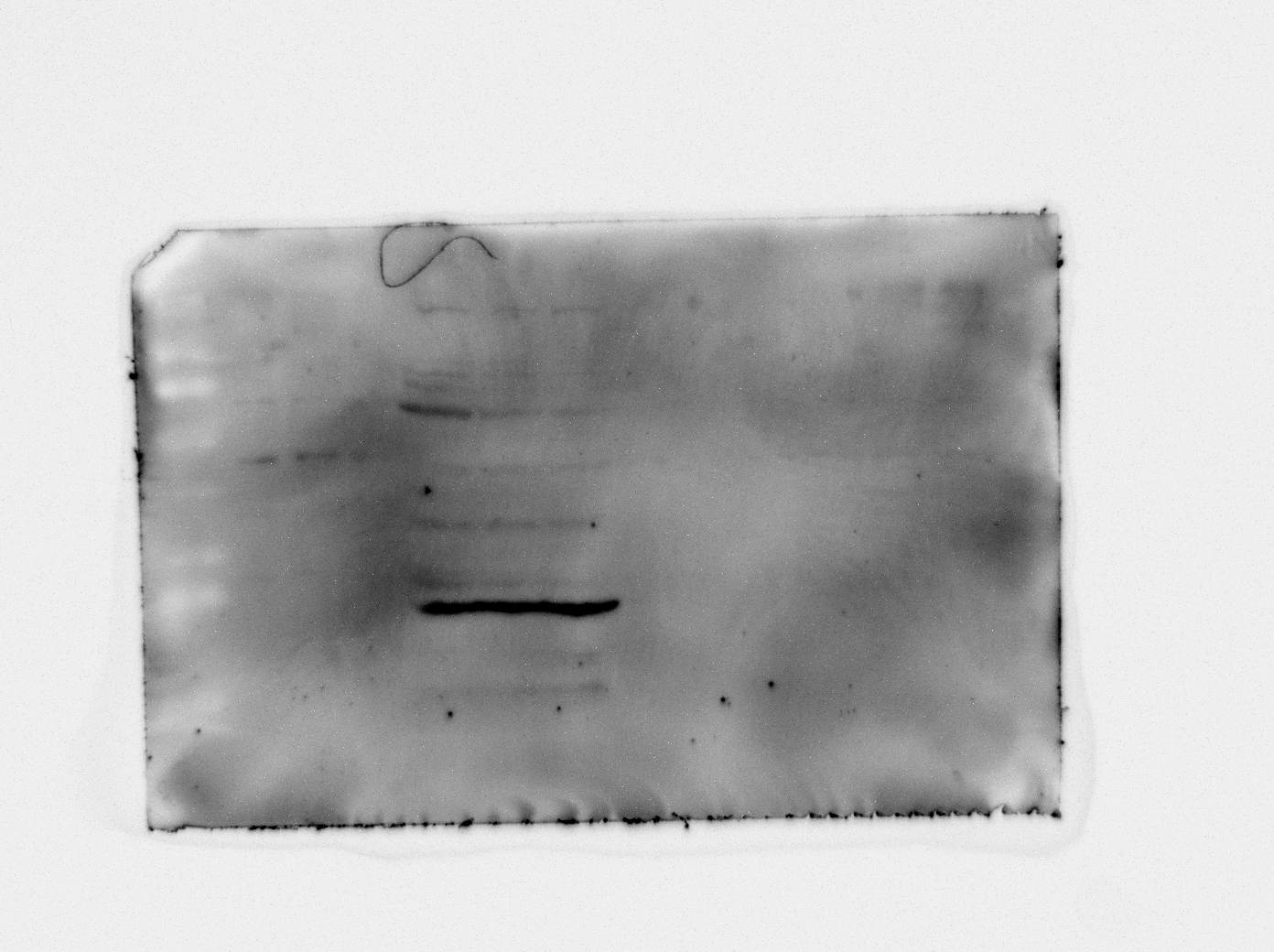

Supplement: Supplementary file 1 [file molecules-27-04985-s001.zip › S5F_TGF-b1.tif]

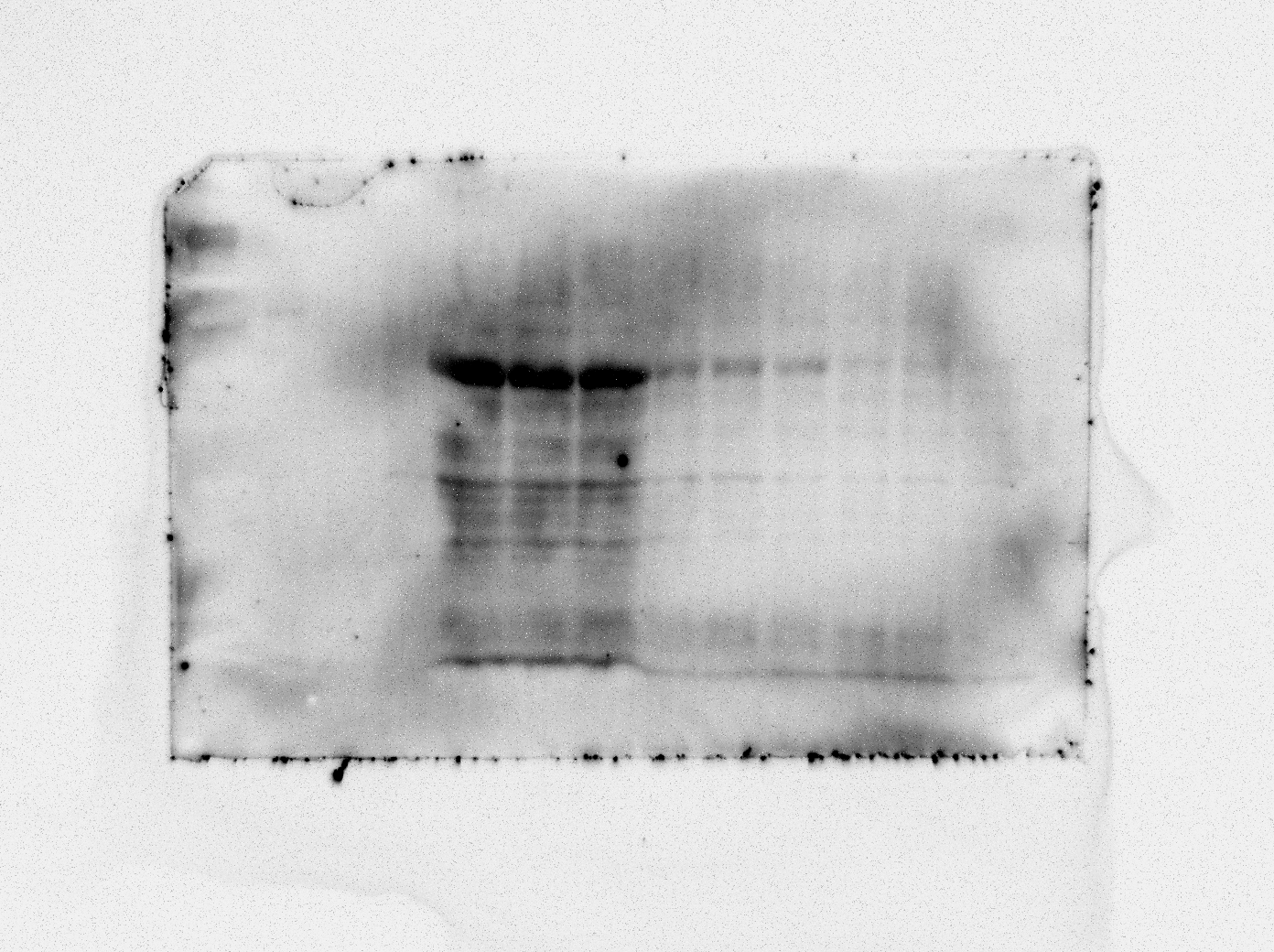

Supplement: Supplementary file 1 [file molecules-27-04985-s001.zip › S5G_a-Tubulin.tif]

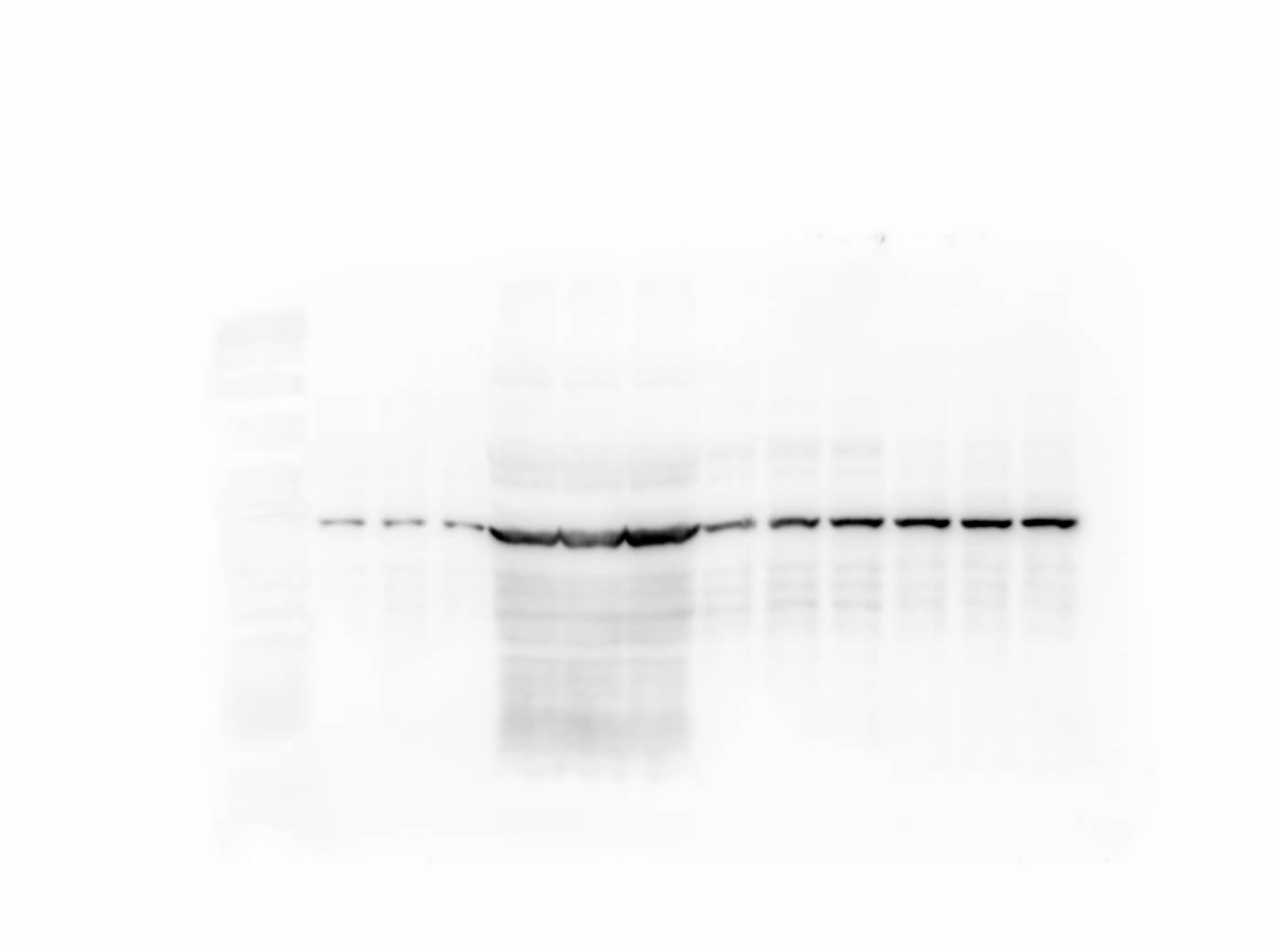

Supplement: Supplementary file 1 [file molecules-27-04985-s001.zip › S5H_Vimentine.tif]

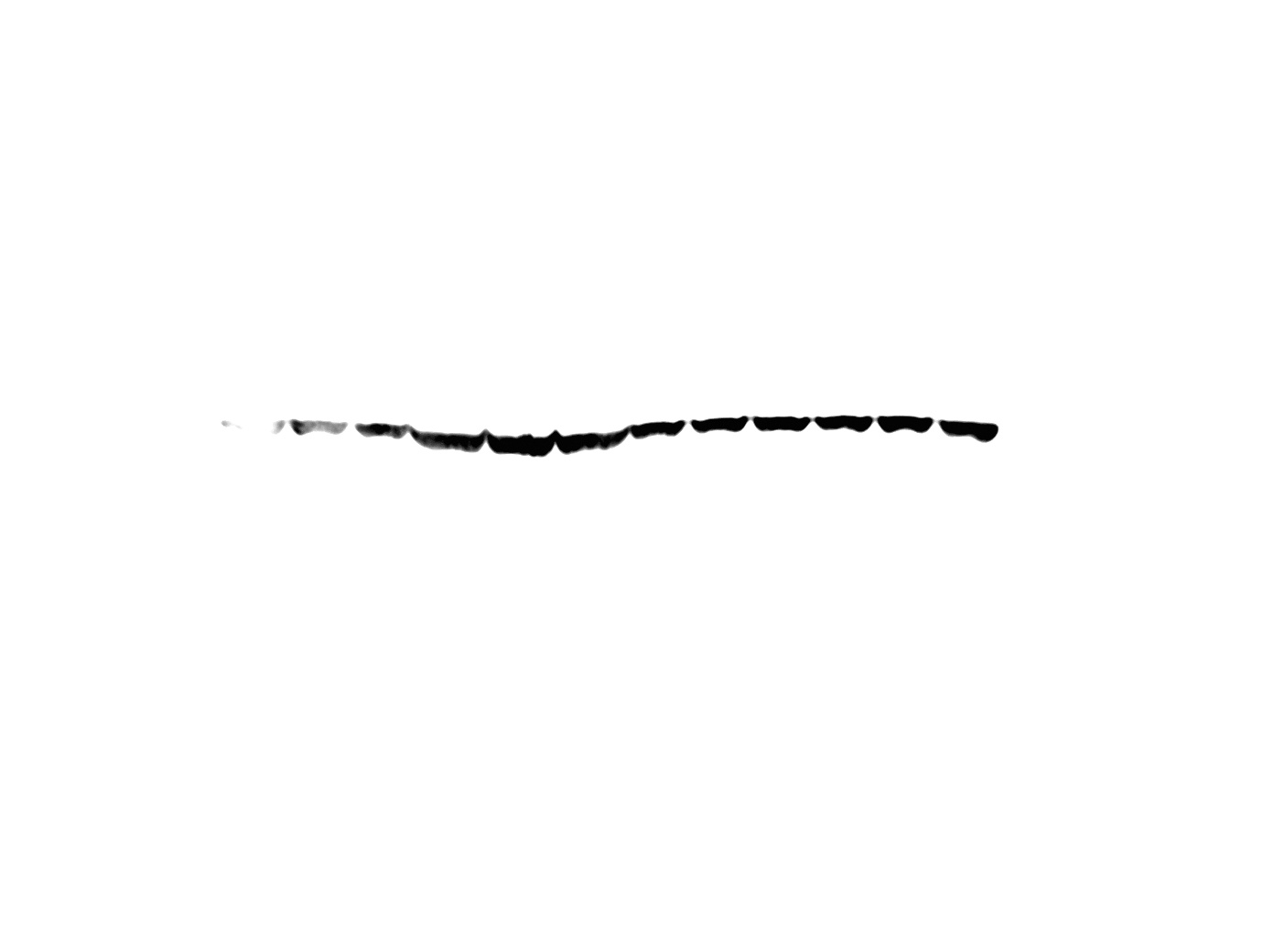

Supplement: Supplementary file 1 [file molecules-27-04985-s001.zip › S5I_Fibronectin.jpg]

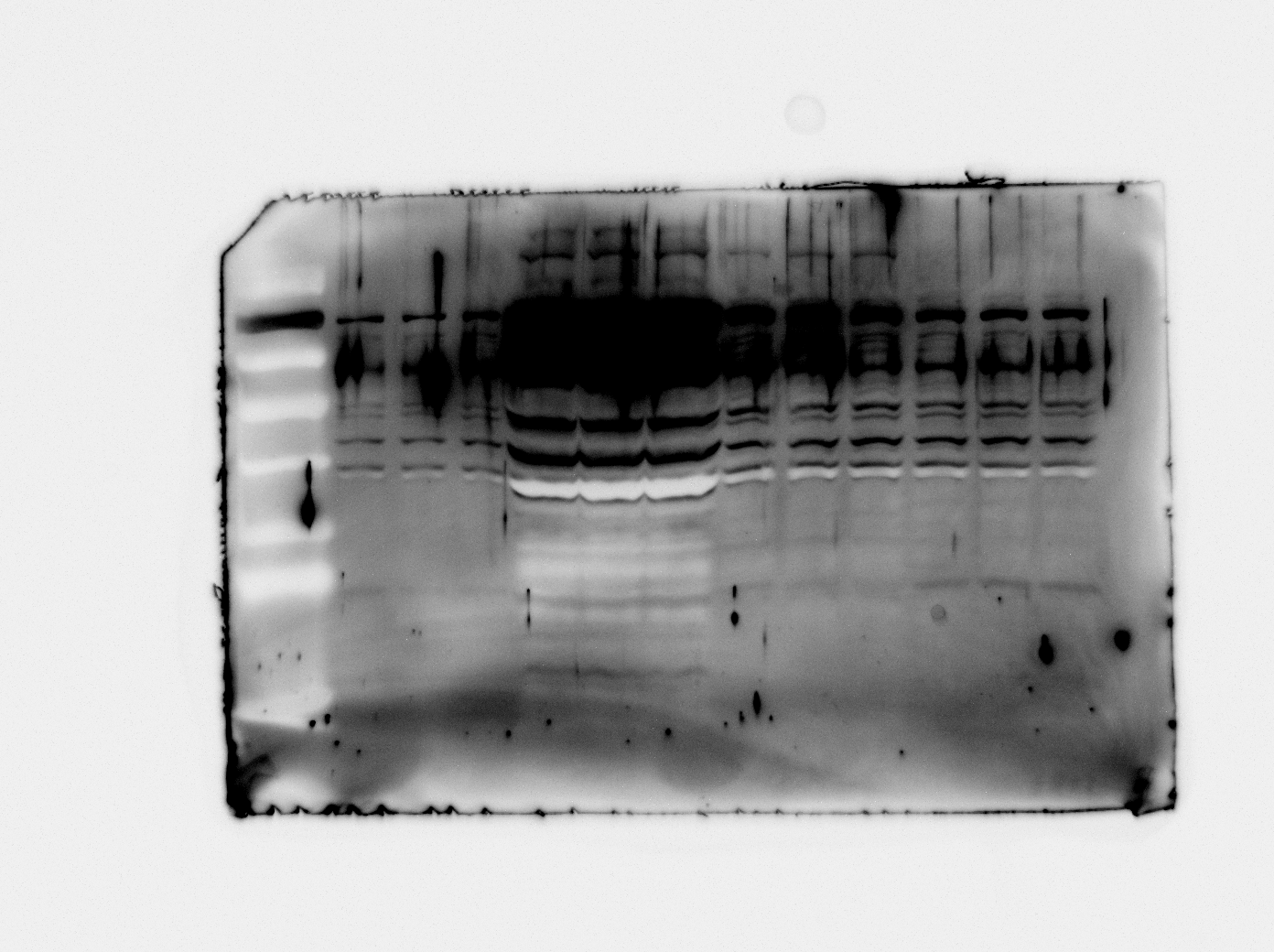

Supplement: Supplementary file 1 [file molecules-27-04985-s001.zip › S5J_Collagen1A.tif]

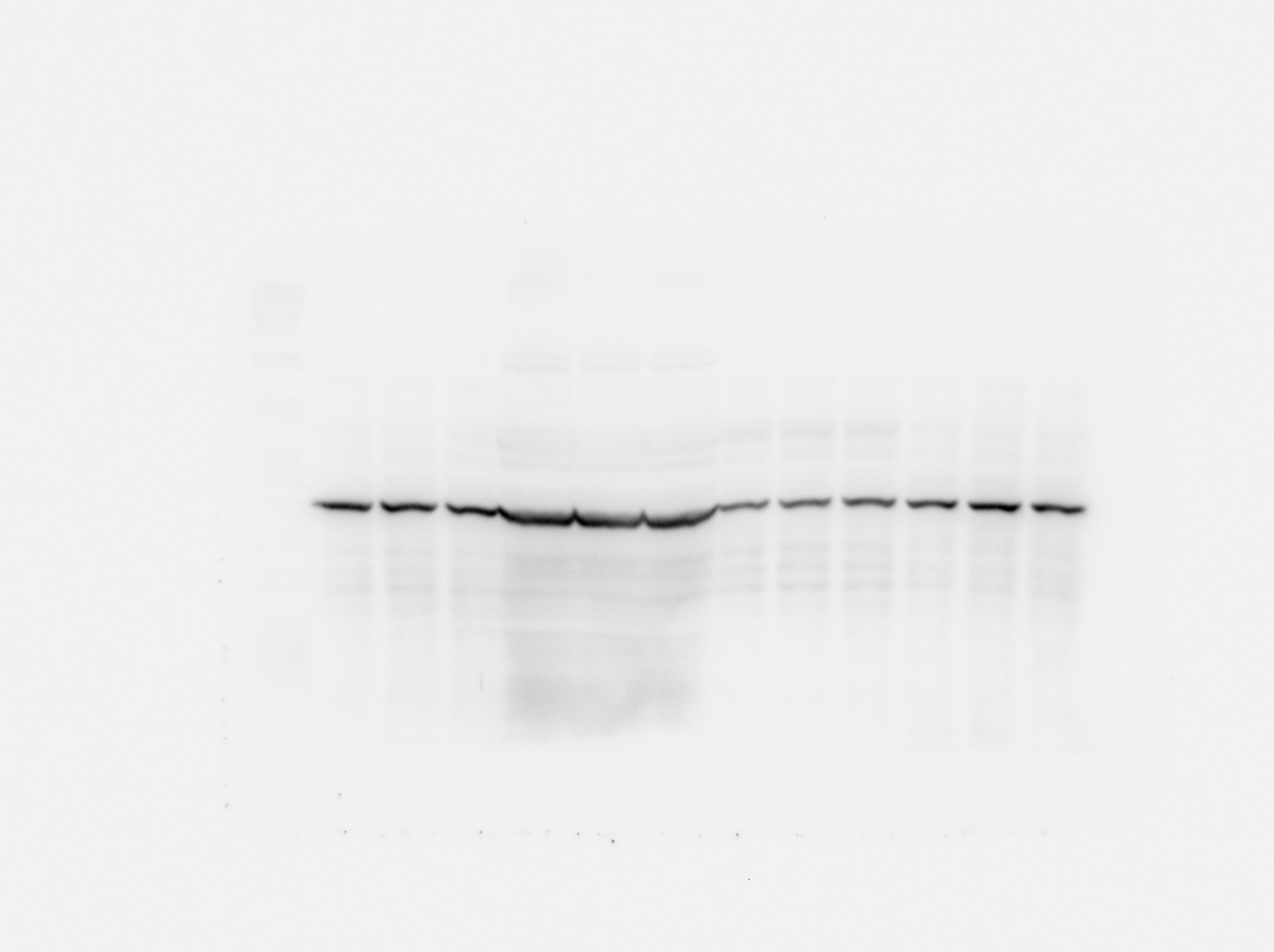

Supplement: Supplementary file 1 [file molecules-27-04985-s001.zip › S5K_a-SMA.tif]

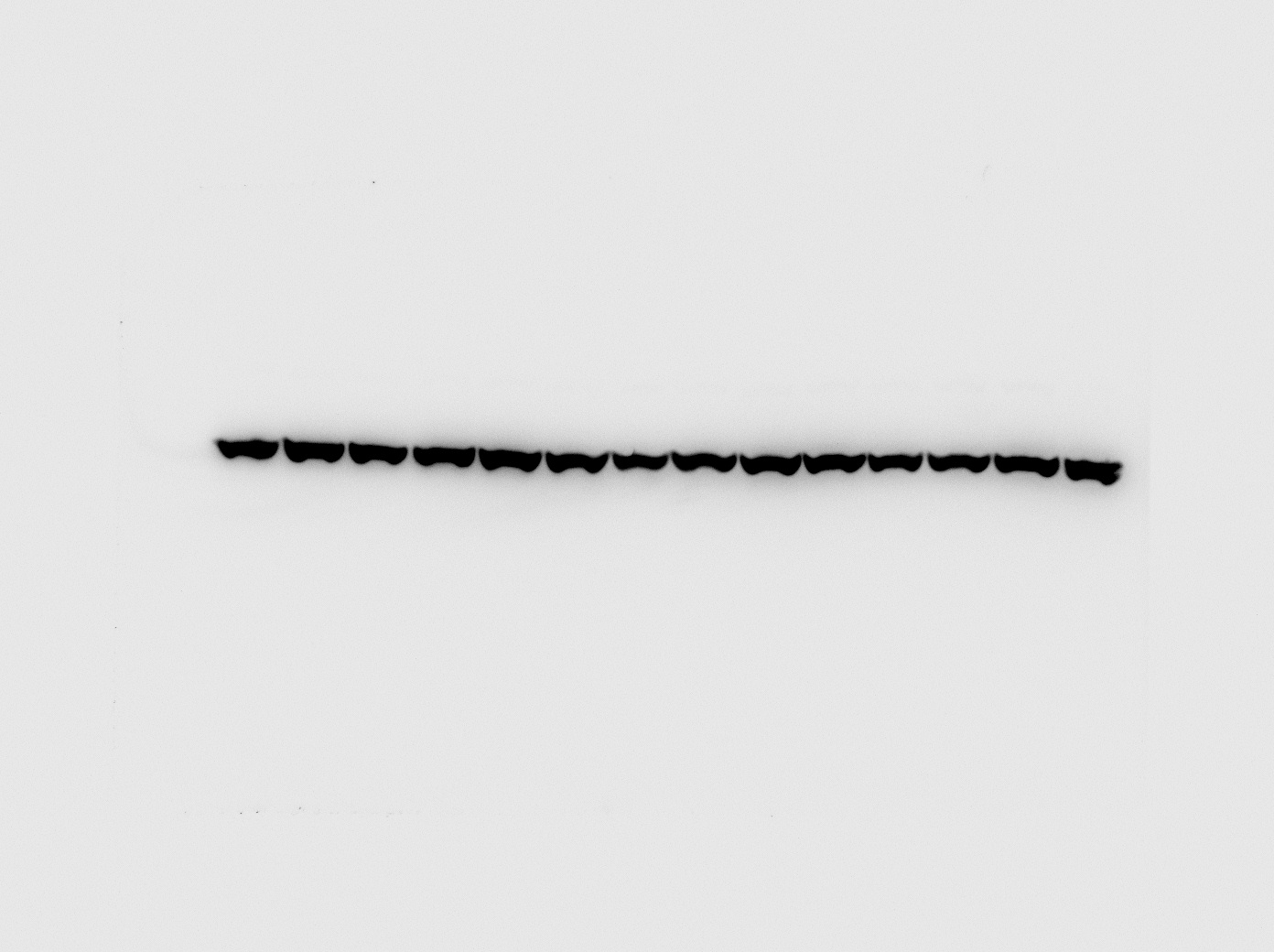

Supplement: Supplementary file 1 [file molecules-27-04985-s001.zip › S5L_b-actin_2.jpg]

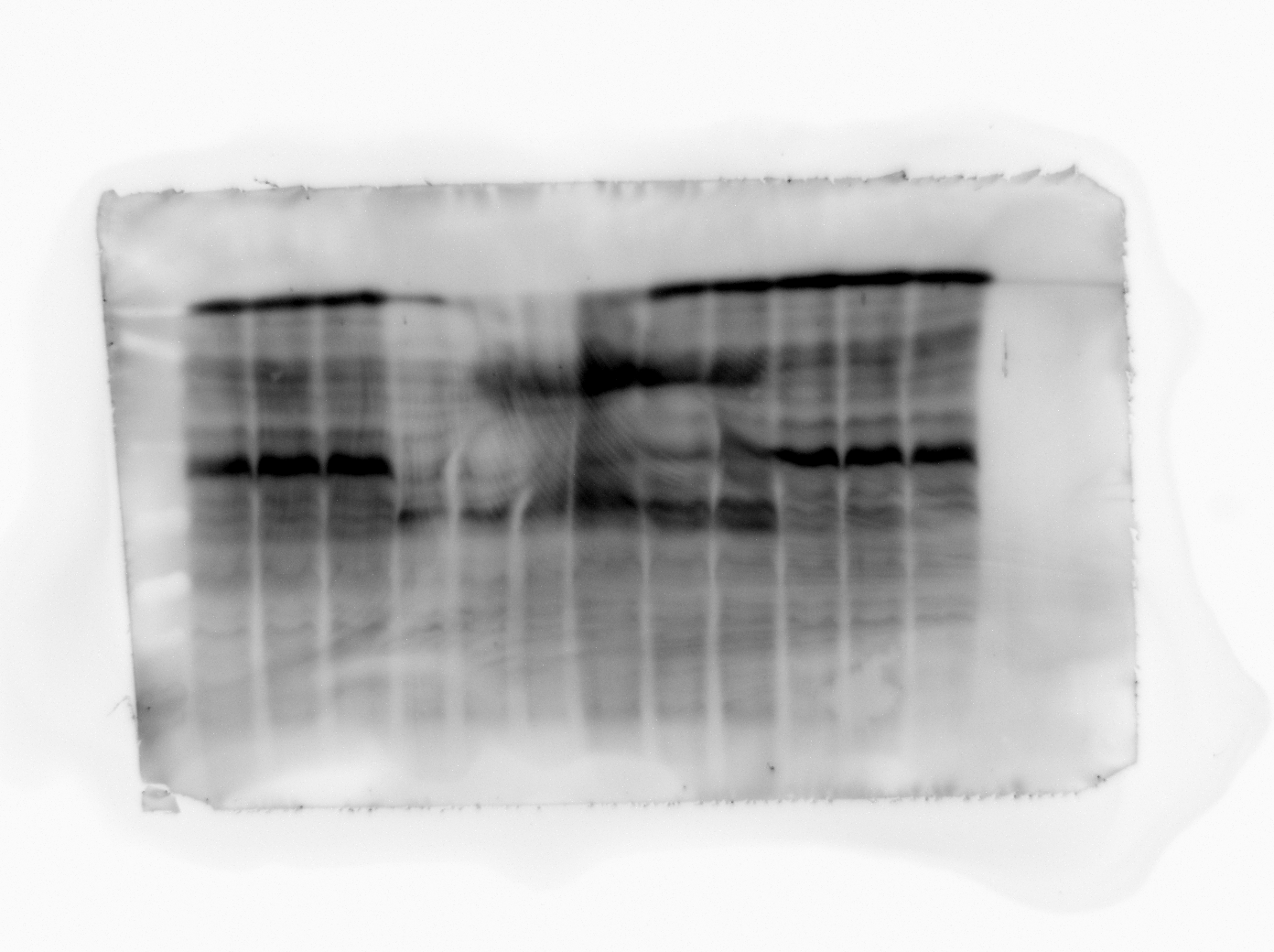

Supplement: Supplementary file 1 [file molecules-27-04985-s001.zip › S5M_SIRT1.tif]

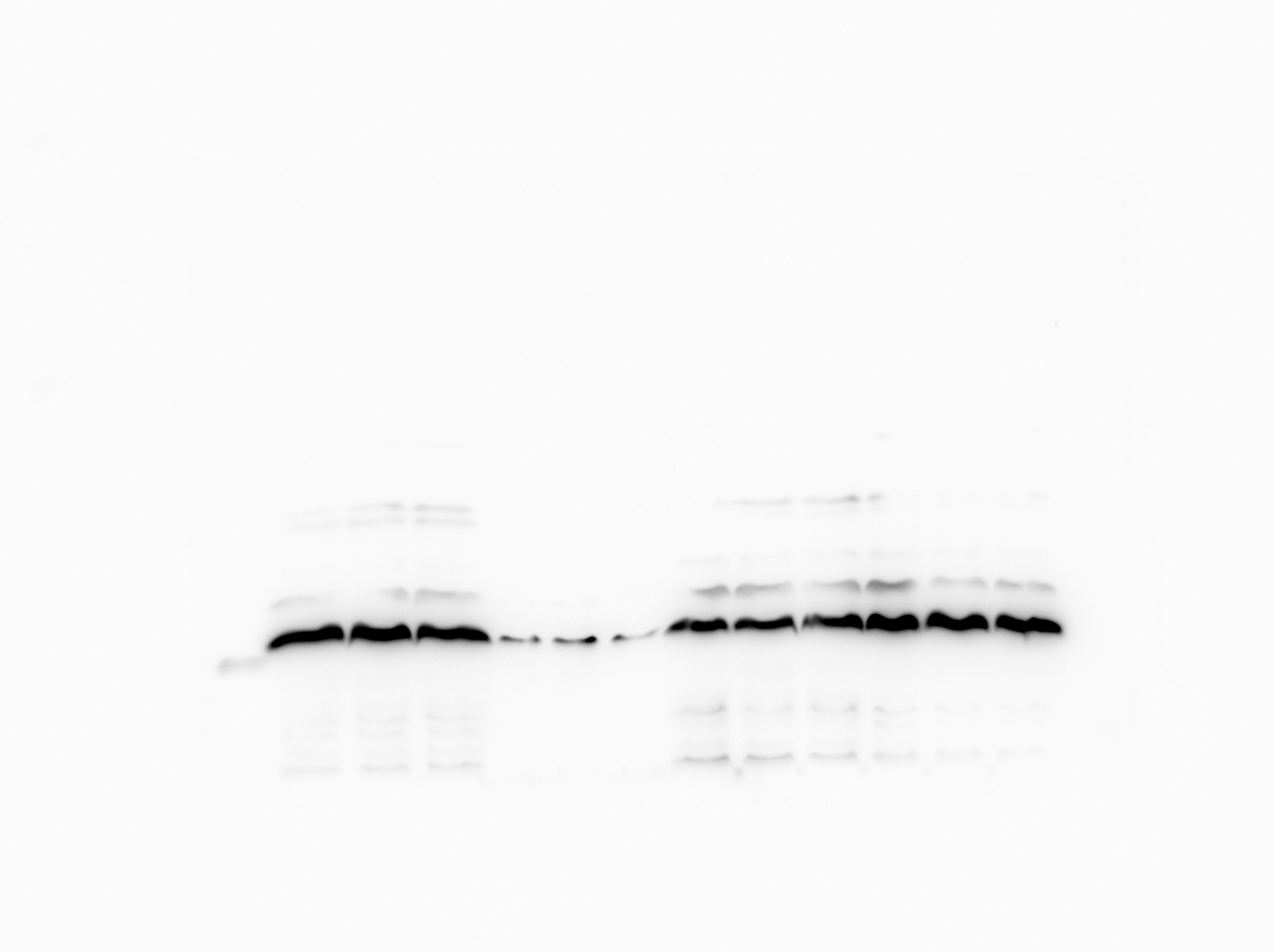

Supplement: Supplementary file 1 [file molecules-27-04985-s001.zip › S5N_SIRT3.tif]

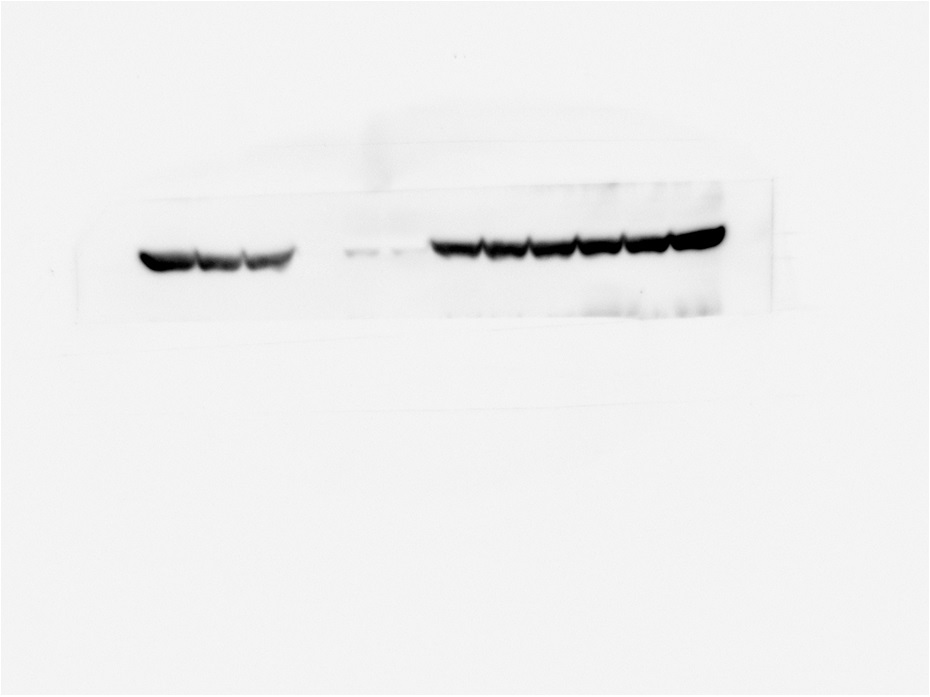

Supplement: Supplementary file 1 [file molecules-27-04985-s001.zip › S5O_SIRT4.jpg]

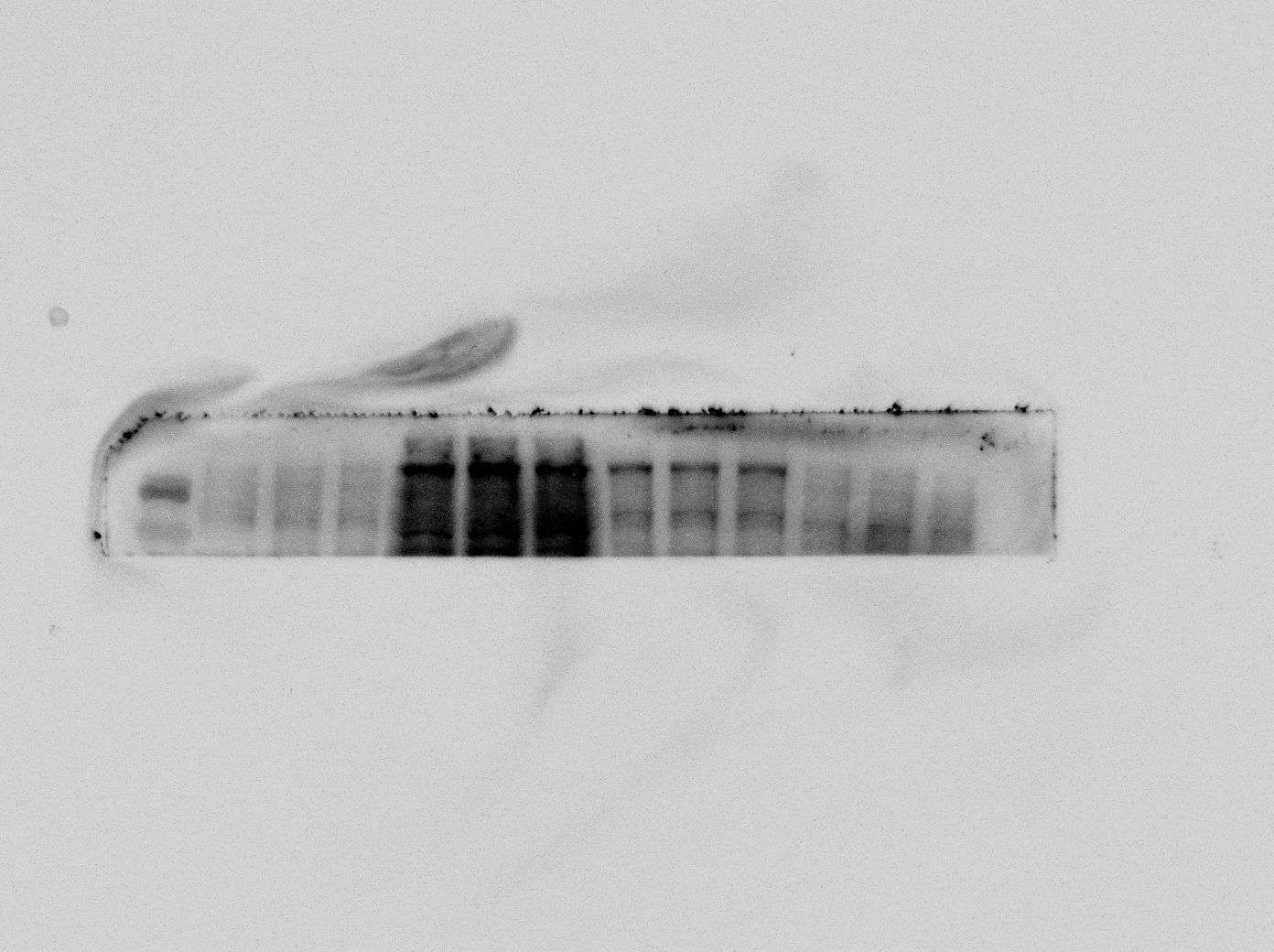

Supplement: Supplementary file 1 [file molecules-27-04985-s001.zip › S5P_Claudin 1.tif]

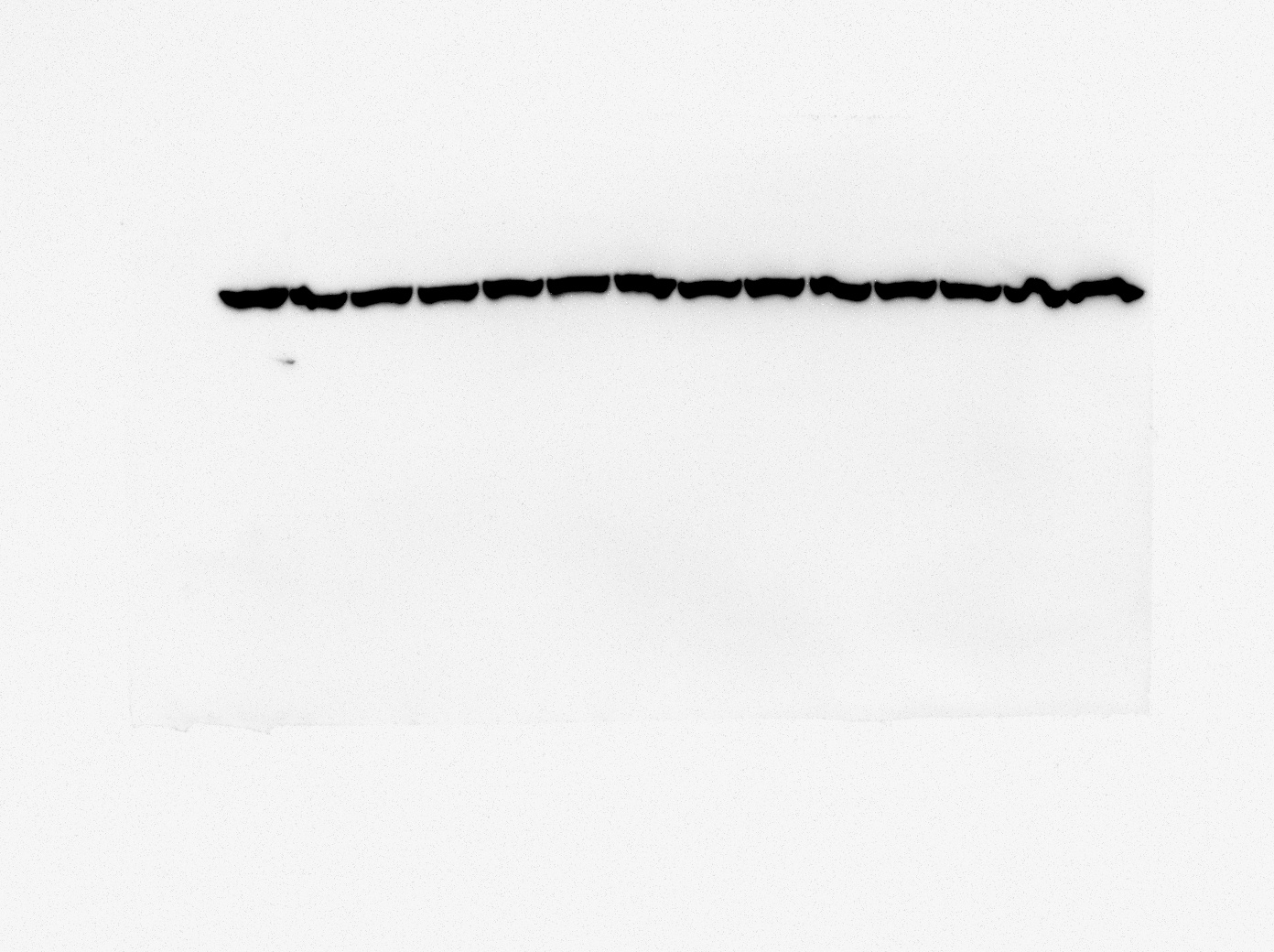

Supplement: Supplementary file 1 [file molecules-27-04985-s001.zip › S5Q_b-actin_1.jpg]

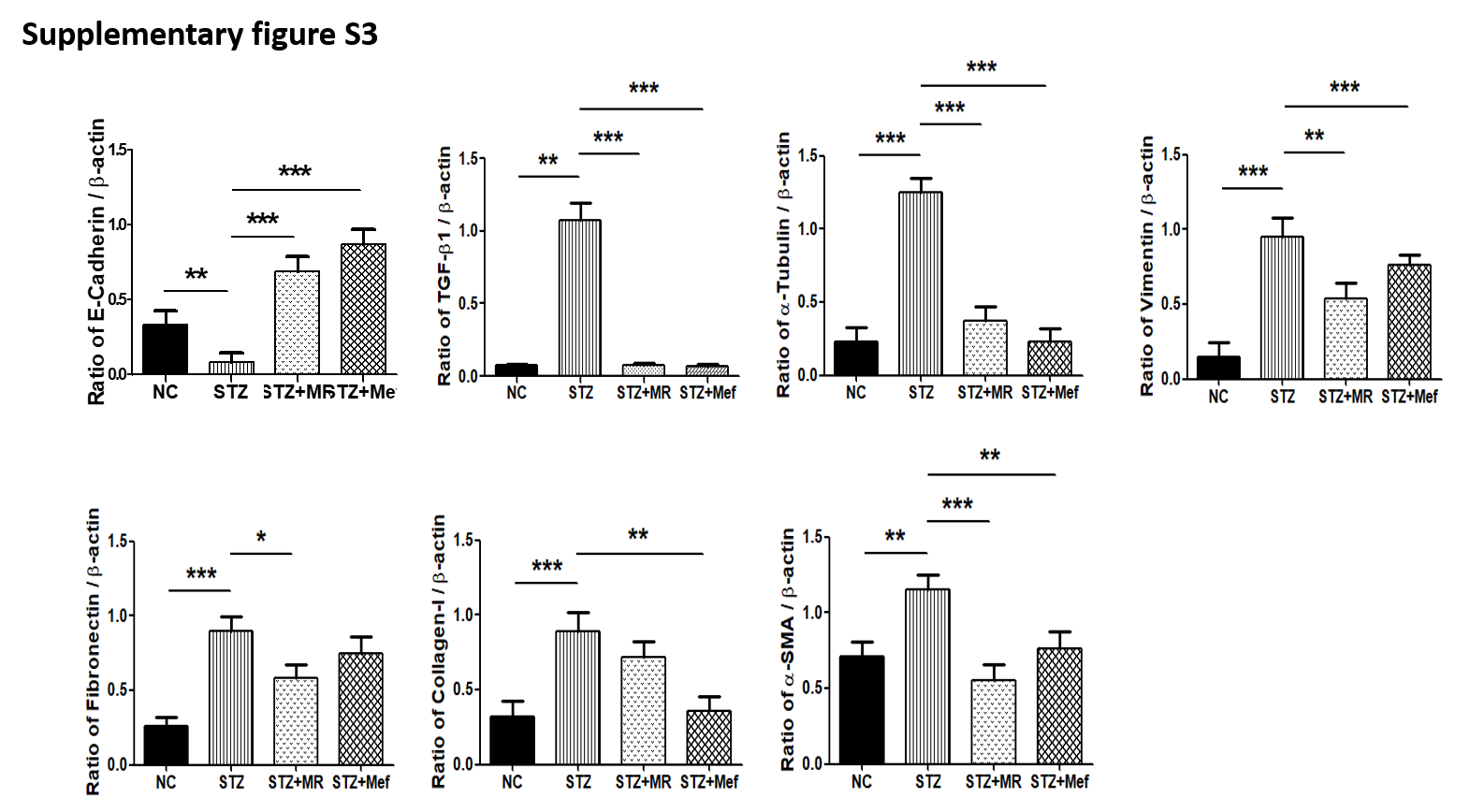

Supplement: Supplementary file 1 [file molecules-27-04985-s001.zip › Supplementary figure S3.tif]

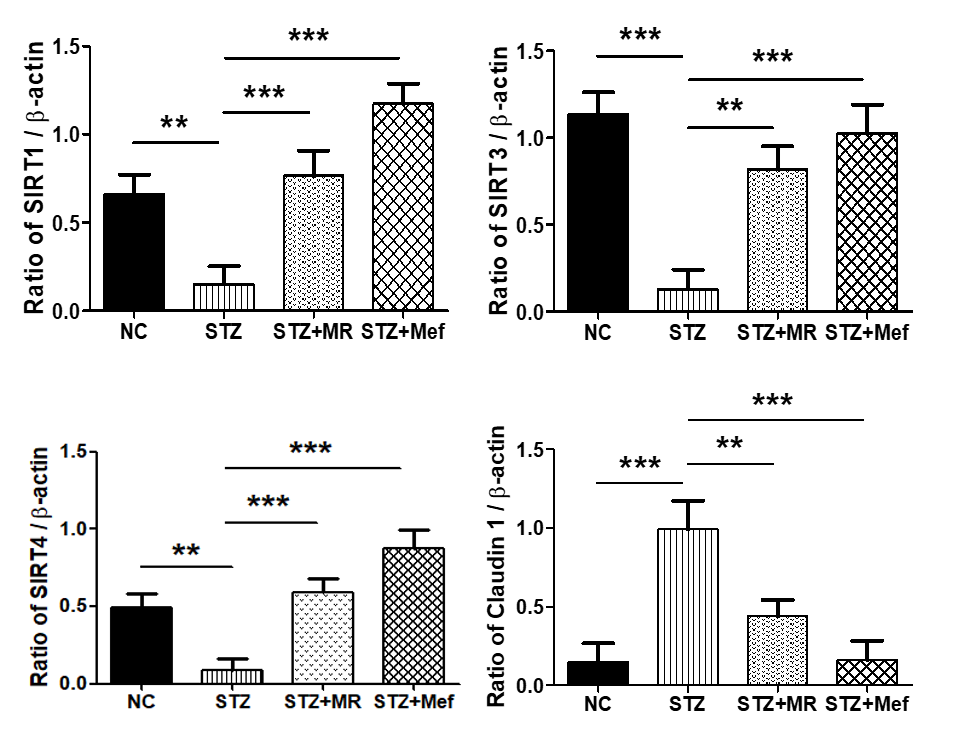

Supplement: Supplementary file 1 [file molecules-27-04985-s001.zip › Supplementary figure S4.tif]
